# Prevalence of gestational diabetes mellitus in Sub-Saharan Africa: a systematic review and meta-analysis

**DOI:** 10.1016/j.xagr.2026.100607

**Published:** 2026-01-18

**Authors:** Ingrid T. Sabbagh, Faheem Seedat, Atsumi Hirose, Alisha N. Wade

**Affiliations:** 1 School of Public Health, Faculty of Medicine (Sabbagh, Hirose), Imperial College London, London, United Kingdom; 2 Centre for Human Genetics, Nuffield Department of Medicine (Seedat), University of Oxford, Oxford, United Kingdom; 3 Nuffield Department of Women's and Reproductive Health (Seedat), University of Oxford, Oxford, United Kingdom; 4 Research in Metabolism and Endocrinology, Department of Internal Medicine, School of Clinical Medicine, Faculty of Health Sciences (Wade), University of the Witwatersrand, Johannesburg, South Africa; 5 MRC/Wits Rural Public Health and Health Transitions Research Unit, School of Public Health (Wade), University of the Witwatersrand, Johannesburg, South Africa; 6 Division of Endocrinology, Diabetes and Metabolism, Perelman School of Medicine (Wade), University of Pennsylvania, Philadelphia, PA

## Abstract

**Background:**

Gestational diabetes mellitus (GDM) is a significant cause of adverse perinatal outcomes and major risk factor for type 2 diabetes in mother and child. Although global prevalence is estimated at 14%, the burden in sub-Saharan Africa remains unclear due to limited data and variable diagnostic protocols. This study aimed to generate a robust estimate of GDM prevalence in sub-Saharan Africa using methodologically comparable studies, and to assess subregional variation.

**Methods:**

We systematically searched Embase, MEDLINE, CINAHL, Global Health, African Journals Online, and African Index Medicus from January 1990 to March 2025 for observational studies of pregnant women in sub-Saharan Africa screened for GDM at ≥24 weeks’ gestation using an oral glucose tolerance test and internationally recognized criteria. Studies using inconsistent, unclear or incomplete diagnostic protocols or self-reported data were excluded. Quality was assessed using the Joanna Briggs Institute checklist. Prevalence estimates were pooled using random-effects meta-analysis of Freeman–Tukey–transformed proportions. Subgroup analyses were conducted by subregion, and mixed-effects meta-regression examined study-level moderators.

**Findings:**

Fifty-nine studies met the inclusion criteria, of which 49 were selected for meta-analysis based on use of comparable diagnostic criteria. Studies represented 16 countries and involved 27,540 participants. The pooled GDM prevalence was 14.0% (95% CI, 11.6 to 16.5; prediction interval 1.9 to 34.3) with substantial heterogeneity (I²=97.1%). Prevalence varied across subregions: Southern Africa 10.2%, Eastern Africa 13.9%, Western Africa 15.1%, and Central Africa 18.0%. Meta-regression showed that small studies (<300 participants), studies using point-of-care testing, and studies conducted before 2016 reported higher prevalence. Subregional differences persisted after adjustment.

**Interpretation:**

When comparable diagnostic protocols are applied, GDM prevalence in sub-Saharan Africa matches the global average, challenging perceptions of a lower regional burden. Subregional variability highlights the need for locally representative data. Standardized diagnostic criteria for epidemiological studies would improve comparability and inform targeted public health interventions.

**AJOG Global Reports at a Glance**

**Why was this study conducted?**

In January 2025 we conducted a PubMed search for systematic reviews with or without meta-analyses on the prevalence of gestational diabetes mellitus (GDM) in Africa or sub-Saharan Africa, using the search terms “gestational diabetes” AND “Africa” with no limitations on language or year of publication. Our search identified 5 systematic reviews published between 2014 and 2024, all of which were limited by small numbers of included countries, variable diagnostic protocols, and insufficient comparable data to allow robust subregional analysis. Notwithstanding the shortage of data, the historical perspective has been that the burden of GDM in Africa (including sub-Saharan Africa) is lower than the global average.

**Key findings**

To our knowledge, this is the most comprehensive systematic review and meta-analysis of GDM prevalence in sub-Saharan Africa to date, and the only 1 rigorously restricted to studies using internationally recognized and methodologically comparable diagnostic criteria. We synthesized data from 49 studies including 27,540 women across 16 countries. The pooled prevalence of GDM was 14.0% (95% CI, 11.6 to 16.5), aligning with the global average. We also identified subregional variation, ranging from 10.2% in Southern Africa to 18.0% in Central Africa, and showed that small sample size and use of point-of-care testing independently inflated prevalence estimates. These results confirm that GDM prevalence in sub-Saharan Africa is comparable to global levels and suggest it has been historically underestimated.

**What does this add to what is known?**

GDM prevalence in sub-Saharan Africa matches the global average, warranting greater attention from policymakers and health systems. Subregional variability underscores the need for locally representative data and context-specific strategies for screening and management. Given the region’s already high perinatal mortality and rising diabetes burden, health authorities should prioritize timely diagnosis and follow-up of women with GDM.

## Introduction

Gestational diabetes mellitus (GDM) is defined as hyperglycemia first detected during pregnancy that does not meet the threshold for overt diabetes mellitus and typically resolves postpartum.^1^ Globally it impacts an estimated 1 in 7 pregnancies^1^ and is associated with an increased risk of adverse perinatal outcomes including macrosomia, preterm delivery, and low Apgar scores.^2^ GDM also increases the long-term risk of type 2 diabetes tenfold in affected women^3^ and fivefold in their offspring.^4^ The prevalence of GDM is projected to rise by 45% between 2024 and 2050, underscoring its significance as a public health concern. Reliable estimates of GDM prevalence are therefore vital to enable health authorities to implement strategies to manage the associated perinatal risks and mitigate long-term cardiometabolic consequences. Given that sub-Saharan Africa has both the highest perinatal mortality rate^5^ and the steepest projected increase in diabetes mellitus prevalence,^1^ a focus on adequate perinatal management of GDM must become a priority.

The accurate reporting of GDM prevalence is complicated by a lack of consensus regarding screening and diagnostic strategies. The most widely accepted criteria to date are captured in the 2010 recommendation by the International Association of Diabetes and Pregnancy Study Group (IADPSG),^6^ which proposed universal one-step testing at 24-28 weeks’ gestation. The threshold values proposed by the IADPSG were based on the landmark "Hyperglycemia and Adverse Pregnancy Outcomes (HAPO)" study^7^ and resulted in GDM detection rates of up to 2 times that of earlier protocols.^8^ Although endorsed by the World Health Organization (WHO) in 2013,^9^ the IADPSG protocol has not been universally implemented. In many settings, resource constraints or concerns over the medicalization of pregnancy have led to continued use of risk-based screening, higher diagnostic thresholds or lower-yielding tests (eg, fasting blood glucose or glycated hemoglobin).^10^

This absence of a standardized diagnostic approach hampers the accurate assessment of the burden of GDM within individual countries or regions and makes comparisons across different settings challenging. In sub-Saharan Africa in particular, comparable data on GDM prevalence are scarce. We identified 5 systematic reviews describing GDM prevalence in Africa or sub-Saharan Africa published between 2014 and 2024,^11^, ^12^, ^13^, ^14^, ^15^ all of which were limited by data scarcity and/or methodological heterogeneity. The earliest 2 reviews published in 2014 and 2015 contained data representing only 6 countries and generated with a variety of protocols, none of which used the IADPSG or comparable criteria.^11^^,^^12^ The 3 later reviews published between 2019 and 2024 had better coverage, representing 11 to 12 countries and containing some data generated using the IADPSG and equivalent criteria. They were, however, still limited by the inclusion of multiple screening and diagnostic protocols, and lacked sufficient comparable data for meaningful subregional analysis.

In this study, our primary aim was to produce a robust estimate of the prevalence of GDM in sub-Saharan Africa by including studies with comparable diagnostic approaches. Our secondary aim was to compare GDM prevalence between sub-Saharan Africa sub-regions.

## Methods

### Search strategy and selection criteria

For this systematic review and meta-analysis, we followed the PRISMA 2020 guidelines^16^ and the Joanna Briggs Institute (JBI) methodology for reviews of prevalence and incidence.^17^

We included primary observational studies of pregnant women residing in World Bank-defined sub-Saharan African countries.^18^ Studies were eligible if they reported GDM screening conducted at ≥24 weeks’ gestation using internationally recognized diagnostic criteria (Supplemental Table S1).^19^ Studies were included only if the entire study sample received an Oral Glucose Tolerance Test (OGTT). We included studies published in peer-reviewed journals in any language from 1990 onwards.

We excluded studies that relied on self-reported data; used mixed or inconsistent diagnostic protocols; excluded 1 or more steps of a protocol; lacked sufficient methodological clarity to determine the protocol used; or were conducted entirely on nonrepresentative samples of the population (eg, specialist referrals or subgroups such as immigrants). Where data were duplicated or published in multiple papers, only the study representing the most complete portion of the dataset was retained.

We searched Embase, MEDLINE and CINAHL as primary sources of peer-reviewed publications. To ensure regional publications were included, Global Health, African Journals Online (AJOL), and African Index Medicus (AIM) were also searched. The 5 prior identified systematic reviews^11^, ^12^, ^13^, ^14^, ^15^ as well as systematic reviews identified in the database searches were screened for relevant publications using citationchaser.^20^

We used search terms “gestational diabetes mellitus,” “GDM,” “hyperglycemia in pregnancy,” and “gestational hyperglycemia” as keywords or MESH terms with truncation where appropriate. We combined the GDM-related terms with sub-Saharan African geographic terms using Boolean operators. The geographic search included individual country names, “sub-Saharan Africa,” “Africa South of the Sahara,” and regional terms such as “Eastern Africa,” “Western Africa,” “Central Africa,” and “Southern Africa.” An example MEDLINE search strategy is provided (Supplemental Table S2). The review protocol was registered on PROSPERO (CRD420251014144)^21^ and the final search conducted on March 19, 2025 by ITS.

Studies were imported into the Covidence software platform where de-duplication was performed automatically. Screening of titles and abstracts and subsequent full text screening was performed independently by ITS and FS with disagreements resolved by discussion. Online document translation tools were used to translate into English if required.

### Data analysis

Data extraction was performed manually by ITS using a structured Excel spreadsheet developed a priori and validated on the first 10 records. Data fields extracted included authors’ names, publication year, study country, study year, sample size, number of cases, sample frame, response rate, study design, mean participant age, screening week of gestation, screening criteria, diagnostic test type, study setting (urban vs rural), risk factors reported, and outcomes reported (Supplemental Text S1). The methodological quality of the studies was independently assessed by ITS and FS using the JBI critical appraisal checklist for prevalence studies,^17^ which includes specific criteria on sampling frames, sampling methods, and population coverage. Studies were grouped into high, medium and low quality based on the scores averaged across reviewers. Studies were not excluded based on quality, but meta-regression was performed to test for any impact of study quality on reported prevalence. Publication bias was not explicitly tested but considered qualitatively as part of our quality assessment, as per recommendations for meta-analysis of proportions.^22^

Data were analyzed using the metafor^23^ package of R (R Foundation for Statistical Computing, Vienna, Austria; version 4.4.2).^24^ We calculated individual study prevalence estimates and corresponding 95% confidence intervals from the study-level case counts and sample sizes. Proportions were transformed using the Freeman-Tukey double arcsine transformation,^25^ and the transformed proportions pooled using random-effects meta-analysis with the DerSimonian-Laird estimator for between-study variance.^26^ Statistical heterogeneity was quantified using the I^2^ statistic, tau2, and Cochran’s Q test. We performed subgroup analysis by country and geographical subregion to show regional prevalence patterns.

We used mixed-effects meta-regression models to investigate the effect of study-level moderators on reported prevalence. Moderators examined included study sample size (categorized as <300, 300 to 699, and ≥700 participants), study date (categorized as pre-2015, 2015-2019, or 2020 and after), testing method (laboratory, point-of-care (POC), or unknown), sample frame, diagnostic criteria, and study quality. Moderators were selected a priori based on literature precedent of their effect on diagnostic accuracy,^27^ sample representativeness or temporal changes in disease prevalence.^28^ To avoid overfitting, moderator variables were categorized where possible to ensure at least 10 studies per examined moderator group. The proportion of between-study variance explained by the model was summarized as R². Pooled prevalence estimates and meta-regression results were back-transformed for interpretation and presented with 95% confidence intervals and prediction intervals. Statistical significance was defined as *P*<.05.

We identified outlying and influential studies using Baujat plots. Potential outliers were re-evaluated for accuracy of data extraction and methodological appropriateness to determine whether to retain.

Sensitivity analyses were conducted excluding study-level moderators that significantly impacted heterogeneity to determine the robustness of observed regional trends.

## Results

**Figure 1:**
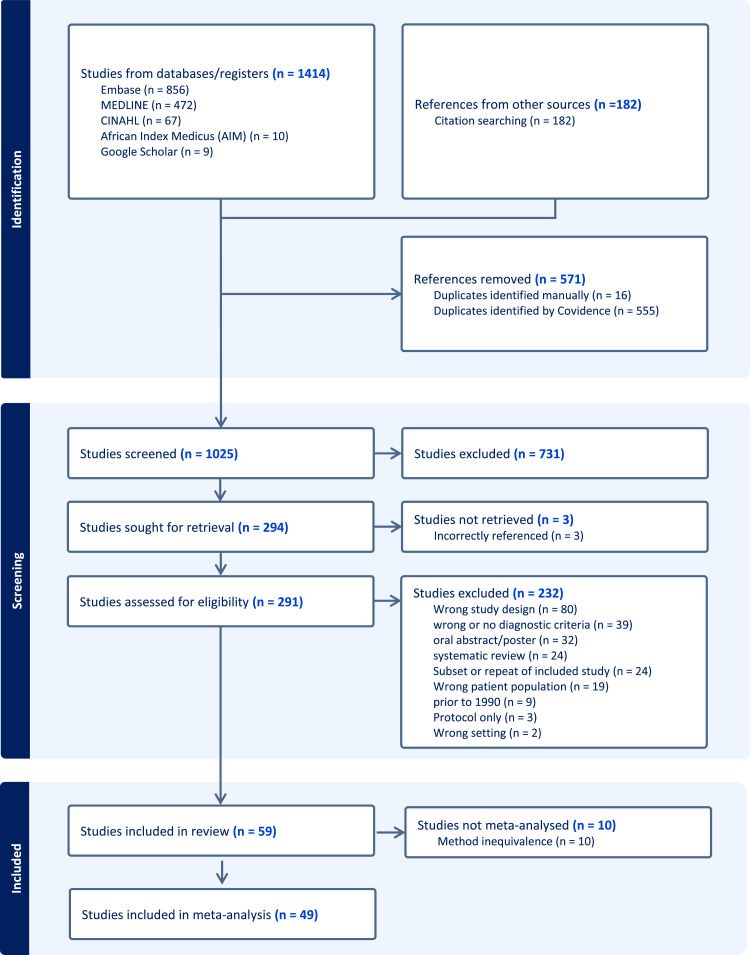
Study selection

Of 1596 records identified, 1025 remained after deduplication (Figure 1). At title and abstract screen, we deemed 731 records irrelevant and identified 294 studies for retrieval, 3 of which could not be sourced due to incorrect citation. We assessed the full text of the remaining 291 studies, of which 59 met our inclusion criteria. Exclusions were mostly due to inappropriate study designs (qualitative or interventional studies not on representative samples or not calculating prevalence) or nonstandard/undisclosed diagnostic criteria.

Forty nine of the 59 selected studies applied the IADPSG, modified IADPSG or WHO2013 protocols (Supplemental Table S1). Because these protocols use the same threshold values and their combined use has been demonstrated not to introduce statistical heterogeneity into a pooled analysis,^8^ our main meta-analysis was restricted to these 49 studies (Supplemental Table S3). We report results of the sensitivity analysis testing the effect of inclusion of the remaining 10 studies in the supplementary material (Supplemental Text S4).

**Figure 2:**
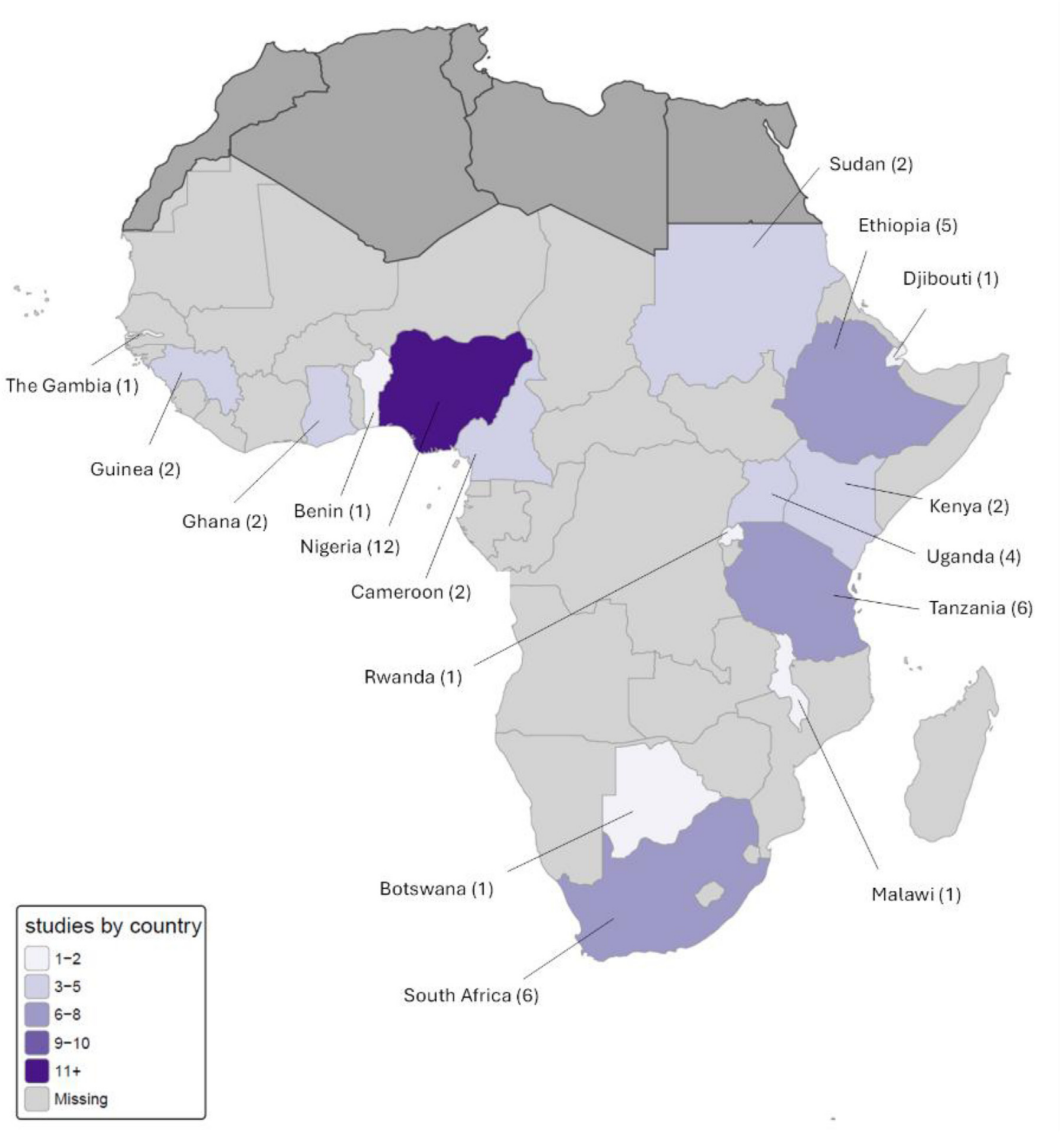
Distribution of included studies by country

The studies included in the meta-analysis were conducted between 2009 and 2023 and involved 27,540 participants from 16 countries representing Eastern, Central, Western and Southern Africa (Table 1, Figure 2). Countries represented were Nigeria^29^, ^30^, ^31^, ^32^, ^33^, ^34^, ^35^, ^36^, ^37^, ^38^, ^39^, ^40^ (12 studies), South Africa^27^^,^^41^, ^42^, ^43^, ^44^, ^45^ and Tanzania^46^, ^47^, ^48^, ^49^, ^50^, ^51^ (6 studies each), Ethiopia^52^, ^53^, ^54^, ^55^, ^56^ (5 studies), Uganda^57^, ^58^, ^59^, ^60^ (4 studies), Cameroon,^61^^,^^62^ Guinea,^63^^,^^64^ Ghana,^65^^,^^66^ Sudan,^67^^,^^68^ and Kenya^69^^,^^70^ (2 studies each), Botswana,^71^ Rwanda,^72^ The Gambia,^73^ Malawi,^74^ Benin^75^ and Djibouti^76^ (1 study each).

**Table 1:** Characteristics of included studies

	Number of Studies	Number of Participants
Subregion		
Central	2	1138
Eastern	22	13,834
Southern	7	5836
Western	18	6732
Sample frame		
Single hospital	22	10,635
Local	12	5848
Multicity	7	6110
Regional	8	4947
Study design		
Cross-sectional	35	18,423
Cohort	13	8829
Case-control	1	288
Protocol		
IADPSG	30	14,804
Modified IADPSG	7	3455
WHO2013	12	9281
Test type		
Laboratory	30	18,026
POC^a^ device	14	7507
Unknown	5	2007
Study year		
2020-2023	10	4611
2016-2019	28	17,782
2009-2015	11	5147
Study size (participants)		
0-299	17	3825
300-699	22	10,180
700+	10	13,535

a POC = point of care.

Sabbagh. Prevalence of gestational diabetes mellitus in Sub-Saharan Africa: a systematic review and meta-analysis. Am J Obstet Gynecol 2026.

The majority of studies were cross-sectional or cohort studies conducted in a single institution or city/village, with relatively few multicity or regional studies. Study sizes ranged from 142 to 2917 participants, with most falling in the range of 300 to 700 participants. Thirty of the studies used laboratory methods for diagnosis, while 14 used POC devices and 5 did not specify an analytical method. Overall study quality was good, with only 10 studies falling in the low- or medium-quality categories. A full list of studies and their characteristics as well as quality assessment results is available in the supplementary material (Supplemental Table S3).

**Figure 3:**
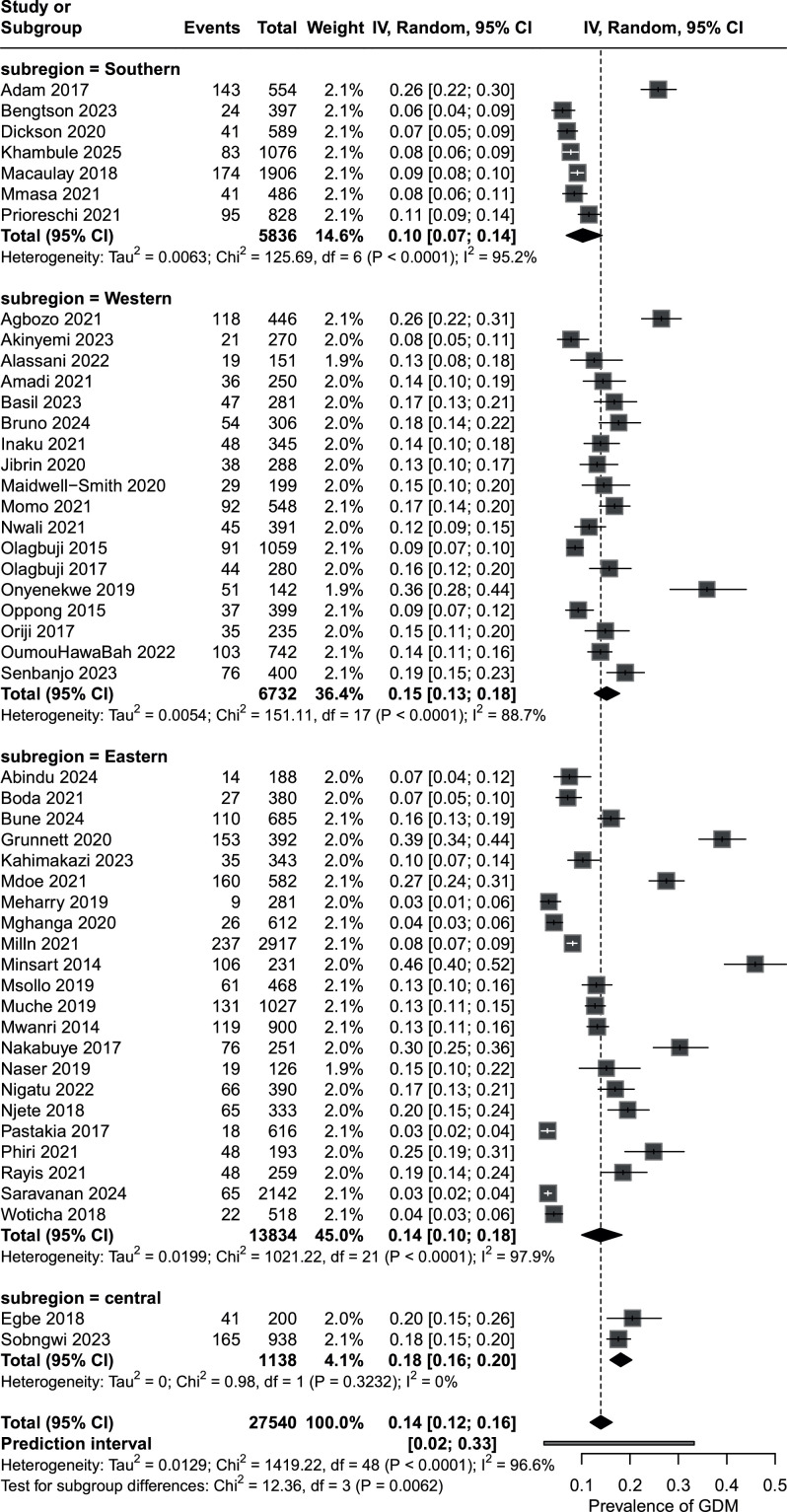
Forest plot of gestational diabetes mellitus prevalence by Sub-Saharan African subregion

The overall pooled prevalence of GDM in the included studies was 14.0% (95% CI, 11.6 to 16.5) with a prediction interval of 1.9% to 34.3% and substantial between-study heterogeneity (I^2^=97.1%). Subregional analysis showed significant differences in GDM prevalence across sub-Saharan Africa (Q_(between)_=12.4 on 3 df, *P*=0.006), summarized in Figure 3. Prevalence was lowest in Southern Africa at 10.2% (6.8 to 14.2); intermediate in Eastern Africa at 13.9% (10.0 to 18.3) and Western Africa at 15.1% (12.6 to 17.9), and highest in Central Africa at 18.0% (15.9 to 20.3), although it must be noted that Central Africa is represented by only 2 studies from a single country.

Sabbagh. Prevalence of gestational diabetes mellitus in Sub-Saharan Africa: a systematic review and meta-analysis. Am J Obstet Gynecol 2026.

In country-level subgroup analysis, prevalence estimates ranged from a low of 3.0% (95% CI, 2.4 to 3.7) in Kenya to a high of 45.9% (95% CI, 39.5 to 52.3) in Djibouti (Supplemental Figure S1). In countries with more than 3 studies, in-country heterogeneity was markedly variable. Ethiopia, Nigeria and South Africa had reasonably consistent findings with absolute between-study variance of less than 10% (τ < 0.1) while results in Tanzania, Uganda and Ghana varied by up to 16% (Supplemental Text S2).

Mixed-effect meta-regression (Table 2) using our proposed moderators explained 18% of between-study heterogeneity, with the omnibus test of moderators reaching significance (QM_11_=21.2, *P*=0.031). Studies of fewer than 300 participants reported higher prevalence than large (>700) studies (β=0.119; 95% CI, 0.027 to 0.212; *P*=0.011). POC testing was associated with higher prevalence than lab-based testing (β=0.108; 95% CI, 0.025 to 0.191; *P*=0.011), and studies conducted prior to 2016 were associated with higher prevalence than those conducted from 2020 onwards (β=0.145; 95% CI, 0.010 to 0.281; *P*=0.036). Sample frame, diagnostic criteria, study age and quality were not significantly associated with prevalence. It should be noted that subregion tested as a moderator did not reach the significance threshold, likely due to high residual heterogeneity and imbalanced subgroup sizes.

**Table 2:** Mixed-effects meta-regression of study-level moderators on gestational diabetes mellitus prevalence

Moderator	β	95% CI	*P*-value
Intercept	0.2608	0.1353 to 0.3864	<.0001^b^
Study size <300 vs ≥700	0.1193	0.0269 to 0.2117	.0114^a^
Study size 300-699 vs ≥700	0.0628	–0.0299 to 0.1556	.1844
Lab test (POC vs lab-based)	0.1078	0.0250 to 0.1906	.0107^a^
Lab test (unknown vs lab-based)	–0.0007	–0.1614 to 0.1600	.9931
Sample frame (local vs single-hospital)	–0.0249	–0.1209 to +0.0712	.6115
Sample frame (multicity/regional vs single-hospital)	–0.0171	–0.1092 to +0.0750	.7160
Criteria (IADPSG-modified vs IADPSG)	–0.1010	–0.2255 to +0.0234	.1116
Criteria (WHO2013 vs IADPSG)	–0.0298	–0.1135 to +0.0538	.4843
Study age (“old” vs recent)	0.0341	–0.0687 to +0.1369	.5152
Study age (“older” vs recent)	0.1455	0.0098 to 0.2812	.0356^a^
Quality (low/medium vs high)	0.0569	–0.0560 to +0.1698	.3231

a *P*<.05.

b *P*<.001.

Examination of our Baujat plot (Supplemental Figure S2) identified 2 studies as disproportionately influential on both heterogeneity and the pooled prevalence estimate a regional study of 392 participants in rural Tanzania^51^ and a single-institution study of 231 participants in Djibouti.^76^ There was potentially some volunteer bias in the Djibouti study and both studies made use of POC devices, but they were both deemed otherwise methodologically sound and therefore retained in the meta-analysis.

In a sensitivity analysis excluding studies of fewer than 300 participants and those conducted using POC tests and prior to 2016, overall pooled prevalence dropped to 10.9% (95% CI, 8.0 to 14.3) but statistical significance of subregional differences remained (Supplemental Text S3). The prediction interval remained wide, but with a reduction of the upper bound from 34% to 28%. These studies were retained in the final analysis to preserve consistency with other global meta-analyses^77^ and enable comparison of our findings with the broader literature.

Sensitivity analysis including the 10 non-IADPSG-equivalent studies resulted in a negligible shift of the overall prevalence to 13.2% (95% CI, 11.1 to 15.4), but meta-regression indicated that both the Carpenter and Coustan and WHO 1985 methods were significant contributors to between-study heterogeneity (Supplemental Text S4).

## Discussion

We identified an overall GDM prevalence of 14.0% (95% CI, 11.6 to 16.5) from 49 studies across 16 countries in sub-Saharan Africa. Prevalence varied markedly by subregion. Meta-regression showed that small study size and use of POC tests independently inflated prevalence estimates and these factors, together with studies conducted prior to 2016, explained 18% of between-study variance. Subregional differences remained robust to these moderators, underscoring the heterogeneity of the GDM burden across the region and highlighting methodological factors that may skew prevalence estimates.

Our pooled prevalence of 14.0% for sub-Saharan Africa is very similar to the global pooled prevalence of 14.7%, calculated by Saeedi et al.^8^ using strictly IADPSG and equivalent diagnostic criteria. It also aligns well with findings by Wang et al.*^77^* in a 2021 study for the International Diabetes Federation (IDF) diabetes atlas, in which they calculated the global GDM prevalence and that of the African region to be 14.2% and 14.3% respectively. It is worth noting that these were unexpected findings at the time, as prior IDF prevalence figures reported for Africa were significantly lower than the global average.^78^^,^^79^ The IDF makes use of empirically derived conversion factors to generate prevalence statistics for their diabetes atlas from studies conducted using different protocols.^1^^,^^79^ In contrast to the IDF approach, our calculation does not rely on conversion factors and combines only results from directly comparable protocols, reducing between-study heterogeneity and likely yielding more reliable pooled estimates. The fact that we see similar results confirms that the GDM burden in sub-Saharan Africa parallels global levels and warrants the same public health focus in the continent as it receives elsewhere.

Muche et al.^13^ also performed a subregional analysis, reporting similar overall prevalence in sub-Saharan Africa and also noting Central Africa as the subregion of highest prevalence. However, trends for the rest of the region were not consistent with ours, possibly due to methodological differences in several included studies (use of fasting blood glucose and non-IADPSG-equivalent methods) and significantly smaller subgroups.

The reasons for the subregional differences observed in our study are unclear. Despite well-documented associations between obesity, type 2 diabetes mellitus and GDM, our observed subregional GDM patterns do not mirror the incidence of adult-onset type 2 diabetes mellitus^80^ or the prevalence of adult obesity.^81^ Variation in maternal age distributions, parity and reproductive patterns, genetic and ethnic susceptibility, urban–rural and socioeconomic gradients, and environmental and dietary factors may however contribute to the differences. It is likely that these factors also drive the residual heterogeneity of GDM prevalence within subregions and even within countries. It is therefore important that regional health authorities do not rely on widely pooled data but understand localized drivers of prevalence.

Analysis of the effect of risk factors on GDM prevalence was beyond the scope of our review, although anecdotally we noted many of the included studies cited known risk factors associated with GDM such as advanced maternal age, obesity, and family history of diabetes. There are, however, notable exceptions, including 2 studies conducted in rural areas in The Gambia^51^ and Tanzania^73^ which showed high GDM prevalence, and which did not report classical risk patterns either in maternal characteristics or rural–urban trends. Rather than regarding such cases as outliers, we suggest that examination of such patterns can provide insights into the heterogeneity of GDM in diverse settings.

Our findings regarding the moderating effect of small study size are not unexpected, as small studies are known to be associated with larger effect sizes and greater heterogeneity.^82^ Likewise, the higher heterogeneity associated with POC tests vs laboratory tests is to be expected given their dependence on regular calibration and systematic differences between capillary and venous blood measurements. This was experimentally verified in 1 of our included studies.^27^

The main strength of our study is the inclusion of only strictly methodologically comparable studies for the primary meta-analysis, which has enabled what we believe is the first direct comparison to similarly derived global data.^8^ The large number of countries represented, facilitated by searching of regional databases and backward citation searching, has enabled statistically robust analysis at a subregional level. An additional strength is the thorough sensitivity analysis that confirmed the reliability of our principal findings.

Several limitations must also be acknowledged. Firstly, while all included studies did attempt to exclude cases of pregestational or overt diabetes, over 70% of type 2 diabetes mellitus in Africa is undiagnosed^1^ and inclusion of these cases may have inflated GDM prevalence estimates in some included studies. Of note, any meta-analysis of GDM in sub-Saharan Africa would be subject to this limitation. Secondly, many of our studies drew on cohorts from single institutions where women presented for antenatal care, potentially introducing selection bias and reducing generalizability. This effect was not apparent in our meta-regression results when testing sample frame as a moderator. However, we do acknowledge that in all but large population-based studies, the study populations typically represent women who seek and access antenatal care. This likely introduces selection bias, particularly in settings with low antenatal care coverage where women with limited access may be underrepresented. Thirdly, some areas (notably in Central Africa) remain under-represented, hampering a comprehensive subregional analysis. Finally, demographic stratification by age and rural vs urban setting would have been beneficial to assess the impact of these important variables on the heterogeneity of our findings. However, many of the included studies did not provide sufficient detail on these factors to allow these analyses.

Despite these limitations, our study makes a valuable contribution to the literature on the prevalence of GDM in sub-Saharan Africa and its subregions. Future estimates would benefit from the universal adoption of a standardized diagnostic approach to enhance generalizability and comparability. The limitations of the universal adoption of the IADPSG criteria for GDM diagnosis for clinical purposes notwithstanding, its use as a standardized diagnostic approach in epidemiological studies has merit. Longitudinal studies in the region are also necessary to understand the impact of GDM in diverse populations with variable access to health care to inform public health responses. Such efforts must also recognize that improved detection alone is insufficient without parallel strengthening of antenatal and maternity services to manage GDM effectively in systems with limited capacity for chronic care.

We have demonstrated that GDM prevalence in sub-Saharan Africa is comparable to the global average but varies substantially between subregions. This underscores the need for locally representative data to inform context-specific screening and management strategies. In resource‐constrained settings where large laboratory‐based studies are not feasible, small cohort studies and POC testing can provide valuable insights—but their tendency to overestimate GDM prevalence must be recognized and accounted for when planning screening programs and allocating resources.

## CRediT authorship contribution statement

**Ingrid T. Sabbagh:** Writing – review & editing, Writing – original draft, Formal analysis, Data curation, Conceptualization. **Faheem Seedat:** Writing – review & editing, Methodology, Formal analysis. **Atsumi Hirose:** Writing – review & editing, Supervision. **Alisha N. Wade:** Writing – review & editing, Supervision.

## Appendix Supplementary materials

Image, application 1

## Footnotes

Supplementary material associated with this article can be found in the online version at doi:10.1016/j.xagr.2026.100607.
