# Supplementary material for: Prevalence of gestational diabetes mellitus in Sub-Saharan Africa: a systematic review and meta-analysis
Source: AJOG Glob Rep. 2026 Jan 18;6(1):100607. doi: 10.1016/j.xagr.2026.100607 (PMC12925211; doi:10.1016/j.xagr.2026.100607)
Supplement: Supplementary file 1 [file mmc1.pdf]

Supplementary material

PAPER: Prevalence of Gestational Diabetes Mellitus in Sub-Saharan Africa: A Systematic Review and Meta-Analysis

AUTHORS: Ingrid T. Sabbagh PhD, Faheem Seedat DPhil , Atsumi Hirose PhD, Alisha N. Wade DPhil

Table of Contents

Table S1: Common diagnostic criteria for gestational diabetes mellitus. 2

Table S2: Full search strategy – Ovid MEDLINE... 3

Text S1: Full list of data items for extraction.. 5

Table S3: Full list of studies included in meta-analysis 6

Table S4 – Studies not included in main meta-analysis 8

Table S5 – Studies excluded at full text review 9

Text S2 – overall and subgroup meta-analysis results 16

Figure S1 – Overall and country subgroup forest plots.. 22

Figure S2 – Baujat plot 24

Text S3 – Sensitivity analysis data excluding sample size <300, use of POC tests and conducted prior to 2016 25

Text S4 – Sensitivity analysis data including non-IADPSG-equivalent diagnostic criteria 28

Table S1: Common diagnostic criteria for gestational diabetes mellitus.

Criteria	Year	OGTT load (g)	Abnormal values	Fasting		1 hour		2 hours		3 hours	
				mmol/L	mg/dL	mmol/L	mg/dL	mmol/L	mg/dL	mmol/L	mg/dL
NDDG*	1979	100	≥2	5·8	105	10·6	190	9·2	165	8·0	145
CC*	1982	100	≥2	5·3	95	10·0	180	8·6	155	7·8	140
WHO 1985	1985	75	≥1	7·8	140	··	··	7·8	140		
WHO 1999	1999	75	≥1	7·0	126	··	··	7·8	140		
DIPSI†	2006	75	≥1	··	··	··	··	7·8	140		
IADPSG	2010	75	≥1	5·1	92	10·0	180	8·5	153	··	··
Modified IADPSG	2010	75	≥1	5·1	92	··	··	8·5	153	··	··
WHO 2013	2013	75	≥1	5·1-6·9	92-125	10·0	180	8·5-11·0	153-199	··	··
NICE	2015	75	≥1	5·6	101	··	··	7·8	140	··	··

NDDG = National Diabetes Data Group; CC = Carpenter & Coustan; WHO = World Health Organization; DIPSI = Diabetes in Pregnancy Study Group India; IADPSG = International Association of Diabetes and Pregnancy Study Groups; NICE = National Institute for Health and Care Excellence; OGTT = oral glucose tolerance test.

* Performed after pre-screening with a 50g glucose challenge test

† Performed unfasted

Table S2: Full search strategy – Ovid MEDLINE

Ovid MEDLINE(R) ALL <1946 to January 06, 2025>

Row	Command	# Results
1	exp Diabetes, Gestational/	19525
2	GDM.mp.	13284
3	(pregnan* adj4 diabet*).mp. [mp=title, book title, abstract, original title, name of substance word, subject heading word, floating sub-heading word, keyword heading word, organism supplementary concept word, protocol supplementary concept word, rare disease supplementary concept word, unique identifier, synonyms, population supplementary concept word, anatomy supplementary concept word]	20698
4	(gestation* adj4 diabet*).mp. [mp=title, book title, abstract, original title, name of substance word, subject heading word, floating sub-heading word, keyword heading word, organism supplementary concept word, protocol supplementary concept word, rare disease supplementary concept word, unique identifier, synonyms, population supplementary concept word, anatomy supplementary concept word]	27789
5	(hyperglyc*emia adj2 pregnan*).mp. [mp=title, book title, abstract, original title, name of substance word, subject heading word, floating sub-heading word, keyword heading word, organism supplementary concept word, protocol supplementary concept word, rare disease supplementary concept word, unique identifier, synonyms, population supplementary concept word, anatomy supplementary concept word]	601
6	1 or 2 or 3 or 4 or 5	42290
7	Angola.mp. or Angola/	2151
8	Benin.mp. or Benin/	5096
9	Botswana.mp. or Botswana/	3634
10	Burkina Faso.mp. or Burkina Faso/	6299
11	Burundi.mp. or Burundi/	1329
12	Cabo Verde/ or Ca* Verde.mp.	944
13	cameroon.mp. or Cameroon/	10691
14	central african republic.mp. or Central African Republic/	1456
15	Chad.mp. or Chad/	1833
16	Comoros.mp. or Comoros/	703
17	Congo*.mp. or Congo/ or "Democratic Republic of the Congo"/	24196
18	Cote d'Ivoire.mp. or Cote d'Ivoire/	4934
19	Ivory Coast.mp.	2004
20	djibouti.mp. or Djibouti/	558
21	Equatorial Guinea.mp. or Equatorial Guinea/	627
22	Eritrea.mp. or Eritrea/	900
23	Eswatini.mp. or Eswatini/	1016
24	Swaziland.mp.	900
25	Ethiopia.mp. or Ethiopia/	36510
26	Gabon.mp. or Gabon/	2615
27	Gambia.mp. or Gambia/	3905
28	The Gambia.mp.	3897
29	Ghana.mp. or Ghana/	19132
30	guinea/ or guinea-bissau/	2452
31	Kenya.mp. or Kenya/	29710
32	Lesotho.mp. or Lesotho/	1158
33	Liberia.mp. or Liberia/	2456
34	Madagascar.mp. or Madagascar/	6826
35	Malawi.mp. or Malawi/	10839
36	Mali.mp. or Mali/	5473
37	Mauritania.mp. or Mauritania/	944
38	Mauritius/ or Mauriti*.mp.	2429
39	Mozambique.mp. or Mozambique/	5502
40	Namibia.mp. or Namibia/	2580
41	Niger/ or Niger.mp.	17267
42	Nigeria.mp. or Nigeria/	50113
43	Rwanda.mp. or Rwanda/	5401
44	Sao Tome.mp.	371
45	Senegal.mp. or Senegal/	9190
46	Seychelles.mp. or Seychelles/	996
47	Sierra Leone.mp. or Sierra Leone/	3542
48	Somalia.mp. or Somalia/	3213
49	South Africa.mp. or South Africa/	70658
50	South Sudan.mp. or South Sudan/	956

51	Sudan/ or Sudan.mp.	12051
52	Tanzania.mp. or Tanzania/	21083
53	Togo.mp. or Togo/	2216
54	Uganda.mp. or Uganda/	24296
55	Zambia.mp. or Zambia/	8614
56	Zimbabwe.mp. or Zimbabwe/	9577
57	"africa south of the sahara"/ or africa, central/ or africa, eastern/ or africa, southern/ or africa, western/	25495
58	7 or 8 or 9 or 10 or 11 or 12 or 13 or 14 or 15 or 16 or 17 or 18 or 19 or 20 or 21 or 22 or 23 or 24 or 25 or 26 or 27 or 28 or 29 or 30 or 31 or 32 or 33 or 34 or 35 or 36 or 37 or 38 or 39 or 40 or 41 or 42 or 43 or 44 or 45 or 46 or 47 or 48 or 49 or 50 or 51 or 52 or 53 or 54 or 55 or 56 or 57	401053
59	6 and 58	465

Text S1: Full list of data items for extraction

- Title
- Authors
- Year of publication
- Country of study
- Subregion
- Year of study completion
- Sample size
- Number of cases
- Sample frame (single hospital, local, multi-city, regional)
- Response rate
- Study design
- Mean age of participants
- Gestational age at screening
- Diagnostic protocol
- Additional protocols (if multiple)
- Test type (lab, point-of-care device (poc), unknown)
- Age group stratification (y/n)
- Demographic (rural, urban, mixed)
- Risk factors reported
- Sequelae reported

Table S3: Full list of studies included in meta-analysis

Study label	Country	Subregion	Year	Size	Cases	Sample frame	Response rate (%)	Design	Mean age	Protocol	Test type	Risk factors	Sequelae	Study Quality
Abindu 2024	Uganda	east	2022	188	14	multi-city	80,7	cross-sectional	25	IADPSGm*	lab	yes		medium
Adam 2017	South Africa	south	2017	554	143	single hospital	55,4	cross-sectional		IADPSG	lab	yes		high
Alassani 2022	Benin	west	2016	151	19	Local		cohort		IADPSG	unknown		yes	low
Agbozo 2021	Ghana	west	2017	446	118	regional	55,3	cohort		IADPSG	lab	yes	yes	high
Akinyemi 2023	Nigeria	west	2017	270	21	multi-city	96,4	cross-sectional	30	IADPSG	lab			high
Amadi 2021	Nigeria	west	2018	250	36	single hospital		cross-sectional	30,8	IADPSGm	lab			medium
Basil 2023	Nigeria	west	2019	281	47	Local	79,8	cross-sectional	29,2	IADPSG	lab	yes		high
Bengtson 2023	South Africa	south	2022	397	24	local	100	cohort	30	IADPSG	unknown			high
Boda 2021	Ethiopia	east	2020	380	27	Local	99	cross-sectional		IADPSG	poc			high
Bruno 2024	Nigeria	west	2020	306	54	single hospital	84,3	cross-sectional	29,1	IADPSG	lab			high
Bune 2024	Ethiopia	east	2023	685	110	Local	92,4	cross-sectional		WHO2013	poc	yes		high
Dickson 2020	South Africa	south	2017	589	41	single hospital	60,8	cross-sectional	27,8	WHO2013	lab	yes		high
Egbe 2018	Cameroon	central	2017	200	41	regional	100	cross-sectional	27,8	IADPSG	lab	yes		high
Grunnett 2020	Tanzania	east	2016	392	153	regional	72,9	cohort		WHO2013	poc			high
Inaku 2021	Nigeria	west	2019	345	48	Local		cross-sectional	28,7	IADPSG	lab			high
Jibrin 2020	Nigeria	west	2020	288	38	single hospital		case-control		WHO2013	lab			medium
Kahimakazi 2023	Uganda	east	2021	343	35	single hospital	38,3	cross-sectional	27,3	IADPSG	poc	yes		high
Khambule 2025	South Africa	south	2023	1076	83	single hospital	94	cross-sectional	35	IADPSG	lab			high
Macaulay 2018	South Africa	south	2017	1906	174	single hospital	52,2	cross-sectional	30	WHO2013	lab	yes		medium
Maidwell-Smith 2020	The Gambia	west	2015	199	29	regional	100	cohort		WHO2013	lab			high
Mdoe 2021	Tanzania	east	2018	582	160	regional	99,3	cross-sectional	26	IADPSG	poc	yes		high
Meharry 2019	Rwanda	east	2017	281	9	multi-city	89,2	cross-sectional		WHO2013	lab			high
Mghanga 2020	Tanzania	east	2017	612	26	single hospital	100	cross-sectional		WHO2013	lab	yes		high
Milln 2021	Uganda	east	2019	2917	237	multi-city	75,7	cohort	27	WHO2013	lab		yes	high

Minsart 2014	Djibouti	east	2014	231	106	single hospital	106	cross-sectional		IADPSG	unknown		yes	medium
Mmasa 2021	Botswana	south	2019	486	41	Local		cohort		IADPSG	unknown			medium
Momo 2021	Guinea	west	2020	548	92	single hospital		cross-sectional	27,05	IADPSG	poc			high
Msollo 2019	Tanzania	east	2018	468	61	regional		cross-sectional	28	IADPSGm	poc	yes		high
Muche 2019	Ethiopia	east	2019	1027	131	Local	92,5	cross-sectional	27,22	IADPSG	poc	yes		high
Mwanri 2014	Tanzania	east	2012	900	119	multi-city	84	cross-sectional		IADPSGm	poc	yes		high
Nakabuye 2017	Uganda	east	2014	251	76	single hospital	75,4	cohort	29,25	WHO2013	poc	yes	yes	high
Naser 2019	Sudan	east	2015	126	19	single hospital	78,7	cohort	27,7	IADPSG	lab			high
Nigatu 2022	Ethiopia	east	2017	390	66	single hospital	92,4	cross-sectional		IADPSG	lab	yes		high
Njete 2018	Tanzania	east	2016	333	65	Local	77	cross-sectional	27,9	WHO2013	poc	yes		medium
Nwali 2021	Nigeria	west	2018	391	45	single hospital	97,8	cross-sectional		IADPSG	lab			high
Olagbuji 2015	Nigeria	west	2012	1059	91	single hospital	82	cross-sectional	30,7	IADPSG	lab	yes		high
Olagbuji 2017	Nigeria	west	2016	280	44	single hospital	100	cross-sectional	30,4	IADPSG	lab			high
Onyenekwe 2019	Nigeria	west	2017	142	51	single hospital	69,3	cross-sectional	28,3	IADPSG	poc			medium
Oppong 2015	Ghana	west	2013	399	37	single hospital		cross-sectional	31	IADPSG	lab	yes		high
Oriji 2017	Nigeria	west	2015	235	35	single hospital	94	cohort		IADPSG	lab	yes		high
OumouHawaBah 2022	Guinea	west	2018	742	103	Local		cross-sectional	28,7	IADPSG	unknown			medium
Pastakia 2017	Kenya	east	2015	616	18	multi-city		cross-sectional	26,1	IADPSG	lab			high
Phiri 2021	Malawi	east	2013	193	48	Local	77,2	cross-sectional	25	IADPSGm	lab	yes		high
Prioreschi 2021	South Africa	south	2016	828	95	Local	81,4	cohort		WHO2013	lab		yes	high
Rayis 2021	Sudan	east	2018	259	48	single hospital	89,3	cohort	28	IADPSG	lab	yes		high
Saravanan 2024	Kenya	east	2019	2142	65	regional	63,4	cohort	26,9	IADPSG	lab	yes		high
Senbanjo 2023	Nigeria	west	2023	400	76	single hospital		cross-sectional	31	IADPSG	lab	yes		high
Sobngwi 2023	Cameroon	central	2009	938	165	multi-city	95,5	cross-sectional	25,5	IADPSGm	poc	yes		high
Woticha 2018	Ethiopia	east	2017	518	22	regional	91,8	cross-sectional	25,7	IADPSGm	poc	yes		high

* IADPSGm = modified IADPSG

Table S4 – Studies not included in main meta-analysis

Study label	Country	Subregion	Year	Size	Cases	Sample frame	Response rate (%)	Design	Mean age	Protocol	Test type	Risk factors	Sequelae
Adegbola 2008	Nigeria	West	2008	222		single hospital	82.8	case-control	31,8	CC	lab	yes	
Adekola 2024	Nigeria	west	2019	234	44	local		cohort		DIPSI	lab		
Adoke 2018	Nigeria	west	2018	207	16	local		cohort	28,2	WHO 1999	lab	yes	
Anzaku 2013	Nigeria	West	2009	253	21	single hospital	95,5	Cross-sectional		DIPSI	Lab		
Jao 2013	Cameroon	central	2013	316	20	Single hospital		Cross-sectional		CC	unknown		
Kuti 2011	Nigeria	west	2009	765	106	Single hospital		Cross-sectional	32,3	WHO 1999	lab	yes	
Larebo 2021	Ethiopia	east	2020	420		regional	89,4	cross-sectional		DIPSI	unknown		
Mamabolo 2006	South Africa	south	2000	262	19	regional		Cross-sectional	25,5	WHO 1999	lab		
Paka 2009	DRC	central	2005	834	43	local		cross-sectional	28,4	CC	unknown	yes	
Seyoum 1999	Ethiopia	east	1999	890	33	Regional	95	Cross-sectional	27	WHO 1985	poc		yes

Table S5 – Studies excluded at full text review

**author, year**	title	**reason**
Adam 2018	Evaluating the utility of a point-of-care glucometer for the diagnosis of gestational diabetes.	Subset or repeat of included study
Adeoye 2022	The Ibadan Pregnancy Cohort Study (IbPCS), a Prospective Cohort Study Protocol	Protocol only
Boerstra 2022	The impact of maternal hyperglycaemia first detected in pregnancy on offspring blood pressure in Soweto, South Africa	Subset or repeat of included study
Chivese 2021	The influence of maternal blood glucose during pregnancy on weight outcomes at birth and preschool age in offspring exposed to hyperglycemia first detected during pregnancy, in a South African cohort.	Wrong patient population
Dias 2019	Prevalence of and risk factors for gestational diabetes mellitus in South Africa	systematic review
Ekwueme 2018	Hyperglycemia and beta cell function in pregnancy	oral abstract
Imoh 2015	Appraisal of timing for oral glucose tolerance testing in relation to risk factors for gestational diabetes mellitus in pregnant women in a Nigerian Teaching Hospital.	no diagnostic criteria specified
Jao 2023	UNIQUE EICOSANOID/LIPID PROFILES IN PREGNANT WOMEN LIVING WITH HIV IN BOTSWANA	oral abstract
Lutale 1993	Glucose tolerance during and after pregnancy in nondiabetic women in an urban population in Tanzania.	no prevalence cited
Macaulay 2018	The effects of gestational diabetes mellitus on fetal growth and neonatal birth measures in an African cohort.	Subset or repeat of included study
Milln 2021	Adverse pregnancy outcomes associated with moderate elevations in blood pressure or blood glucose in Ugandan women; a prospective cohort study.	Subset or repeat of included study
Ozumba 2004	Diabetes mellitus in pregnancy in an African population.	Wrong study design
Tola 2024	Epidemiology of hyperglycemia during pregnancy in Ethiopia: prevalence, associated factors, and feto-maternal outcomes: systematic review and meta-analysis.	systematic review
Abbey 2018	First Trimester Fasting Blood Glucose as a Screening Tool for Diabetes Mellitus in a Teaching Hospital Setting in Nigeria	Wrong patient population
Abdullahi 2016	Serum adiponectin levels in pregnant women with gestational diabetes mellitus in Zaria, north west Nigeria: a cross sectional study	Wrong study design
Abebe 2017	Diabetes in Ethiopia 2000-2016-Prevalence and related acute and chronic complications; a systematic review	systematic review
Abejirinde 2019	Viability of diagnostic decision support for antenatal care in rural settings: findings from the Bliss4Midwives Intervention in Northern Ghana.	Wrong study design
Abera 2024	Double burden of gestational diabetes and pregnancy-induced hypertension in Ethiopia: A systematic review and meta-analysis of observational studies.	systematic review
Abera 2024	Prevalence and Predictors of Gestational Diabetes Mellitus in Sub-Saharan Africa: A 10-Year Systematic Review.	systematic review
Abudu 1987	Screening for diabetes in pregnancy in a Nigerian population with a high perinatal mortality rate.	prior to 1990
Adam 2017	Selective Screening Strategies for Gestational Diabetes: A Prospective Cohort Observational Study.	Subset or repeat of included study
Adam 2017	Comparing screening strategies for gestational diabetes in a South African population	Subset or repeat of included study
Adam 2018	Association between gestational diabetes and biomarkers: a role in diagnosis.	Subset or repeat of included study
Adefisan 2020	Diagnostic accuracy of random plasma glucose and random blood capillary glucose in detecting international association of diabetes and pregnancy study groups- defined hyperglycemia in early pregnancy.	Wrong patient population
Adegbola 2014	Response to fifty grams oral glucose challenge test and pattern of preceding fasting plasma glucose in normal pregnant Nigerians	Subset or repeat of included study
Adeoye 2025	Associations of macrosomia with sociodemographic, anthropometric, lifestyle factors and perinatal outcomes in Southwest Nigeria.	Wrong study design
Adoyo 2016	Retrospective cohort study on risk factors for development of gestational diabetes among mothers attending antenatal clinics in Nairobi County.	Wrong study design
Agbozo 2017	Are we missing pregnant women with gestational diabetes? Evidence from a diagnostic accuracy study comparing glycosuria, glycated haemoglobin, random and fasting glucose to oral glucose tolerance test	oral abstract
Agbozo 2018	Accuracy of glycosuria, random blood glucose and risk factors as selective screening tools for gestational diabetes mellitus in comparison with universal diagnosing.	Subset or repeat of included study
Agbozo 2018	Maternal morbidities in Ghana: Risk factors and effect on newborn health outcomes	oral abstract
Agbozo 2019	isk factors for gestational diabetes and comparison of associated pregnancy outcomes by diagnostic criteria	oral abstract
Agbozo 2022	IDF21-0680 Non-adherence to appointments for gestational diabetes testing and pregnant women[StQuote]s experiences with 2-hour OGTT	no prevalence cited
Agofure 2019	Prevalence of gestational diabetes mellitus among pregnant women attending antenatal care services in Diette Koki memorial hospital, Opolo Bayelsa state, Nigeria	Wrong study design

Ajayi 2015	Prevalence of gestational diabetes using 50 gram glucose challenge test 1 hour result in 1204 cases in Lagos	oral abstract
Akinola 2024	Association of maternal insulin resistance with neonatal insulin resistance and body composition/size: a prospective cohort study in a sub-Saharan African population.	Wrong patient population
Akpan 1989	A comparison of maternal and cord blood glucose levels in diabetic and non-diabetic Nigerians in relation to birth weight and maternal body mass index.	prior to 1990
Al-Shafei 2021	Maternal early pregnancy serum level of 25-Hydroxyvitamin D and risk of gestational diabetes mellitus.	Wrong study design
Alshareef 2018	Helicobacter pylori infection, gestational diabetes mellitus and insulin resistance among pregnant Sudanese women.	Subset or repeat of included study
Amegah 2022	Vitamin D intake modifies the association of household air pollution exposure with maternal disorders of pregnancy.	no diagnostic criteria specified
Anozie 2019	Pregnancy outcome among elderly primigravidae: A five-year review at Abakaliki, Ebonyi state, Nigeria	no diagnostic criteria specified
Argaw 2021	Preterm Premature Ruptures of Membrane and Factors Associated among Pregnant Women Admitted in Wolkite Comprehensive Specialized Hospital, Gurage Zone, Southern Ethiopia.	no diagnostic criteria specified
Asare-Anane 2014	Risk Factors for Gestational Diabetes Mellitus among Ghanaian Women at the Korle-Bu Teaching Hospital	Wrong study design
Atiba 2017	Maternal Plasma Lipid Profile in Women Screened for Gestational Diabetes Mellitus (GDM)	Wrong study design
Atlaw 2022	Incidence and risk factors of gestational diabetes mellitus in Goba town, Southeast Ethiopia: a prospective cohort study.	fasting blood glucose only
Awofisoye 2019	Glycated haemoglobin and obstetric outcomes among patients with gestational diabetes mellitus: a single center study	oral abstract
Ayalew 2019	Prevalence of pre-eclampsia and associated factors among women attending antenatal care services in Felege-Hiwot referral hospital, Bahir Dar city, Northwest Ethiopia	no diagnostic criteria specified
Ayfokru 2023	Incidence and Predictors of Mortality among Neonates Admitted for Congenital Heart Disease in Public Comprehensives Specialized Hospitals, Amhara Region, Ethiopia 2023	Wrong patient population
Aynalem 2020	Incidence of respiratory distress and its predictors among neonates admitted to the neonatal intensive care unit, Black Lion Specialized Hospital, Addis Ababa, Ethiopia.	Wrong patient population
Aytenew 2019	PREVALENCE AND OUTCOME OF PREGESTATIONAL DIABETES MELLITUS AMONG PREGNANT MOTHERS ATTENDING ANTENATAL CARE AT THREE TEACHING HOSPITALS IN ADDIS ABABA, PROSPECTIVE FOLLOW UP STUDY	Wrong patient population
Azeez 2021	A systematic review and meta-analysis of the prevalence and determinants of gestational diabetes mellitus in Nigeria.	systematic review
Basil 2022	Sex Hormone-Binding Globulin Level Enhances Prediction of Gestational Diabetes Mellitus in a Sub-Saharan African Population.	Subset or repeat of included study
Basil 2024	A first trimester prediction model and nomogram for gestational diabetes mellitus based on maternal clinical risk factors in a resource-poor setting.	Subset or repeat of included study
Basu 2010	Obesity and its outcomes among pregnant South African women.	Wrong study design
Bawah 2019	Gestational diabetes mellitus and obstetric outcomes in a Ghanaian community.	Wrong study design
Bawah 2019	Leptin, resistin and visfatin as useful predictors of gestational diabetes mellitus.	Wrong study design
Bawah 2019	First trimester zonulin levels and adiposity as predictive indices of gestational diabetes mellitus	own diagnostic criteria
Belay 2020	Adverse birth outcome and associated factors among diabetic pregnant women in Ethiopia: Systematic review and meta-analysis.	systematic review
Belay 2021	Macrosomia and its predictors in pregnant women with diabetes in Ethiopia.	systematic review
Beyene 2023	Gestational diabetes mellitus and its associated factors in Ethiopia: a systematic review and meta-analysis.	systematic review
Beyuo 2015	Metformin versus Insulin in the Management of Pre-Gestational Diabetes Mellitus in Pregnancy and Gestational Diabetes Mellitus at the Korle Bu Teaching Hospital: A Randomized Clinical Trial.	Wrong study design
Biadgo 2019	Gestational diabetes mellitus in HIV-infected pregnant women: A systematic review and meta-analysis	systematic review
Boadu 2022	Prevalence and Risk Factors Associated With Gestational Diabetes Mellitus Among Pregnant Women: A Cross-Sectional Study in Ghana.	fasting blood glucose only
Boerstra 2021	The impact of hyperglycemia first detected in pregnancy on offspring blood pressure in Soweto, South Africa	oral abstract
Boiro 2017	Newborns of diabetic mothers in the neonatology department of the Dakar University Hospital (Senegal)	Wrong study design
Bouhsain 2009	Etude critique des pratiques de dépistage du diabète gestationnel d'un service de gynécologie obstétrique	Wrong setting
Bune 2024	Gestational Diabetes Mellitus Risk Factors in Pregnant Women Attending Public Health Institutions in Ethiopia's Sidama Region: An Unmatched Case-Control Study	Wrong study design
Challis 2002	Gestational diabetes mellitus and fetal death in Mozambique: an incident case-referent study.	own diagnostic criteria
Chijioke 2019	Seasonality in hyperglycemic emergencies in a Health Facility in Sub-Saharan Africa: The roles of geographic location, infection, and knowledge of diabetes mellitus status	Wrong patient population

Chionuma 2022	Profile of insulin resistance of pregnant women at late third trimester in Nigeria: A descriptive cross-sectional report.	fasting blood glucose only
Chirenje 1992	The effects of established and gestational diabetes on pregnancy outcome at Harare Maternity Hospital.	own diagnostic criteria
Chukwunyere 2015	Gestational Diabetes in a Tertiary Healthcare Centre at Abeokuta, South Western Nigeria: A Five Year Retrospective Review	Wrong study design
Coetzee 1979	Diabetes newly diagnosed during pregnancy: A 4-year study at Groote Schuur Hospital.	prior to 1990
Coetzee 2022	Hyperglycemia First Detected in Pregnancy in South Africa: Facts, Gaps, and Opportunities.	no prevalence cited
Coetzee 2023	Pregnancy and diabetic ketoacidosis: fetal jeopardy and windows of opportunity	Wrong study design
Cutland 2024	Obstetric and neonatal outcomes in South Africa.	no diagnostic criteria specified
Daponte 1999	Management of diabetic pregnant patients in a tertiary center in the developing world.	Wrong patient population
Diop 2015	Diabetes mellitus in sub-Saharan Africa: Epidemiological and socioeconomic aspects	systematic review
Djomhou 2016	Maternal hyperglycemia during labor and related immediate post-partum maternal and perinatal outcomes at the Yaounde Central Hospital, Cameroon.	Wrong study design
Doi 2022	Unifying the diagnosis of gestational diabetes mellitus: Introducing the NPRP criteria.	Wrong study design
Druye 2024	Self-management interventions for gestational diabetes in Africa: a scoping review	Wrong study design
Ekekwe 2012	Perinatal mortality and associated risk factors at Lagos University Teaching Hospital	poster
Ekoh 2023	Investigating Serum Ferritin Levels and Gestational Diabetes Mellitus, in two Healthcare Institutions in Delta State, Nigeria	Wrong study design
Emmanuel 2023	Prevalence And Factors Associated with Post-Caeserean Section Wound Sepsis in A Hospital in Ghana (A Retrospective Audit)	no diagnostic criteria specified
Eshetu 2019	Birth Outcomes among Diabetic Mothers Who Delivered in Tikur Anbessa Specialized Hospital, Addis Ababa, Ethiopia.	no diagnostic criteria specified
Ewnetu 2017	Effects of Level of Glycaemic Control in Reduction of Maternal and Perinatal Complications Among Pregnant Diabetic Women at Tikur Anbessa Specialized Hospital, Addis Ababa, Ethiopia	Wrong study design
Ezegwui 2011	Fetal macrosomia: obstetric outcome of 311 cases in UNTH, Enugu, Nigeria.	Wrong study design
Ezenwaka 1998	Maternal plasma glycaemia and fetal outcome in north-eastern Nigeria	Wrong study design
Feleke 2018	Determinants of gestational diabetes mellitus: a case-control study.	Wrong study design
Feleke 2021	Maternal and newborn effects of gestational diabetes mellitus: A prospective cohort study	Wrong study design
Feleke 2022	Maternal and newborn effects of gestational diabetes mellitus: A prospective cohort study.	Wrong study design
Fernandez-Alonso 2010	Serum 25-hydroxy-vitamin d levels during the first trimester of pregnancy and perinatal outcomes	poster
Firisa 2021	Prevalence of Hypertensive Disorders among Pregnant Women Attending Antenatal Care in Selected Public Hospitals in Addis Ababa, Ethiopia	self-reported
Gaffur 2022	Association between first birth caesarean delivery and adverse maternal-perinatal outcomes in the second pregnancy: a registry-based study in Northern Tanzania.	no diagnostic criteria specified
GarciaVilaplana 2020	Description of Maternal Morbidities Amongst 1000 Women During Pregnancy in Ambanja, Madagascar - Opportunities and Challenges of Using an mHealth System.	fasting blood glucose only
Hall 2011	Diabetes in Sub Saharan Africa 1999-2011: epidemiology and public health implications. A systematic review.	systematic review
Halla 2015	Diabetes mellitus in pregnancy, still changing	Wrong study design
Hassan 2021	Blood Groups and Hematological Parameters Do Not Associate with First Trimester Gestational Diabetes Mellitus (Institutional Experience).	Subset or repeat of included study
Heslehurst 2022	Association between maternal adiposity measures and adverse maternal outcomes of pregnancy: Systematic review and meta-analysis	systematic review
Hinneh 2022	Barriers to screening, diagnosis and management of hyperglycaemia in pregnancy in Africa: A systematic review	systematic review
Huddle 2005	Audit of the outcome of pregnancy in diabetic women in Soweto, South Africa, 1992-2002	Wrong study design
Imoh 2015	Diagnosis of gestational diabetes mellitus by American diabetes association's and World Health Organization's criteria and outcome in pregnant Nigerian women	oral abstract
IssaSouleymane 2024	Prevalence and Risk Factors of Gestational Diabetes in the Antenatal Clinic of the Community Health Center of Sibiribougou: Prévalence et Facteurs de Risque du Diabète Gestationnel en Consultation Prénatale au Centre de Santé Communautaire de Sibiribougou	fasting blood glucose only
Iyoke 2013	Retrospective cohort study of the effects of obesity in early pregnancy on maternal weight gain and obstetric outcomes in an obstetric population in Africa.	Wrong study design
Jackson 1979	Glycosuria as an Indication for Glucose Tolerance Testing during Pregnancy	prior to 1990
Jao 2021	Lower insulin sensitivity early in life with in utero HIV/ART exposure in Botswana	oral abstract
Jiwani 2011	Gestational diabetes mellitus: results from a survey of country prevalence and practices	Wrong study design

John 2015	Foeto-maternal outcome of diabetes in a tertiary health facility in Nigeria	Wrong study design
John 2015	Feto-maternal outcome of diabetes mellitus in pregnancy at the university of port-harcourt teaching hospital, Nigeria	Wrong study design
John 2016	Foeto-maternal outcome of diabetes in a tertiary health facility in Nigeria	Wrong patient population
Kamanu 2008	Fetal macrosomia in African women: a study of 249 cases	Wrong study design
Khambule 2022	Glycated haemoglobin and fasting glucose levels are sensitive markers for gestational diabetes in the Black African population	oral abstract
Khan 2016	Physical activity and the risk for gestational diabetes mellitus amongst pregnant women living in Soweto: a study protocol.	Protocol only
Kiiza 2020	Frequency and Factors Associated with Hyperglycaemia First Detected during Pregnancy at Itojo General Hospital, South Western Uganda: A Cross-Sectional Study.	Wrong study design
Kipkemoi 2024	Socio-medical Factors Associated with Neurodevelopmental Disorders on the Kenyan Coast.	no diagnostic criteria specified
Kisindja 2022	Prevalence of gestational diabetes in Eastern Democratic Republic of Congo.	fasting blood glucose only
Kolozali 2024	Explainable Early Prediction of Gestational Diabetes Biomarkers by Combining Medical Background and Wearable Devices: A Pilot Study With a Cohort Group in South Africa.	Wrong study design
Kumah 2024	Gestational glucose intolerance among pregnant women at the Cape Coast Teaching Hospital.	Wrong patient population
Larbi 2000	Pregnancy outcomes in urban black South African women aged 35 years and older	no diagnostic criteria specified
Lawal 2016	Vitamin D status of women with risk factors for gestational diabetes mellitus in Abuja, Nigeria	oral abstract
Lendoye 2015	Placental malaria and gestational diabetes mellitus impact on fetal growth in Gabon	Protocol only
Lendoye 2022	Prevalence and factors associated to gestational diabetes mellitus among pregnant women in Libreville: a cross-sectional study.	own diagnostic criteria
Lester 1985	Pregnancy in Ethiopian diabetic women.	prior to 1990
Lokrou 1990	Prevalence of gestational diabetes in Cote-d'Ivoire	oral abstract
Lombaard 2009	Do risk factors help to identify women from Africa with gestational diabetes mellitus?	poster
Lombaard 2009	Perinatal mortality due to diabetes mellitus recorded with the Perinatal Problem Identification Program (PPIP) in South Africa	oral abstract
Macaulay 2014	Gestational diabetes mellitus in Africa: a systematic review.	systematic review
Macaulay 2017	The prevalence of gestational diabetes amongst women living in Soweto, South Africa	oral abstract
Magadla 2019	Incidence of hypoglycaemia in late preterm and term infants born to women with diabetes mellitus	Wrong study design
Maidwell-Smith 2018	Hyperglycaemia in pregnancy in Sub-Saharan Africa: An estimate of the prevalence in rural Gambia and the diagnostic ability of capillary glucose versus venous plasma glucose sampling	Subset or repeat of included study
Maidwell-Smith 2019	Hyperglycaemia first detected in pregnancy in sub- Saharan Africa: An estimate of the prevalence in rural Gambia and the diagnostic ability of capillary blood glucose versus venous plasma glucose sampling	Subset or repeat of included study
Makuyana 2005	Occurrence of diabetogenic changes in pregnancy among black women in an urban setting.	fasting blood glucose only
Malaza 2023	Maternal serum adiponectin levels are associated with obesity and diabetes in pregnancy	reference incorrect
Manga 2023	Glycaemic characteristics and maternal and neonatal outcomes in antenatal patients with pre-gestational and gestational diabetes attending the Charlotte Maxeke Johannesburg Academic Hospital, South Africa	oral abstract
Mapira 2017	Strategy to improve the burden of gestational diabetes in African women: Rwandan perspective	own diagnostic criteria
Marais 2016	Screening for gestational diabetes: Examining a breakfast meal test	Wrong study design
Marais 2018	Randomized cross-over trial comparing the diagnosis of gestational diabetes by oral glucose tolerance test and a designed breakfast glucose profile.	Wrong study design
Marconi 2018	Pregnancy outcome in women with diabesity	Wrong patient population
Marie 2015	Does maternal race influence the short-term variation of the fetal heart rate? An historical cohort study.	Wrong setting
Martin 2018	Microalbuminuria among Newly Diagnosed Diabetic Patients at Mulago National Referral Hospital in Uganda: A Cross Sectional Study.	Wrong patient population
May 2025	Hypertensive disorders in a gestational diabetes cohort from Cape Town, South Africa	Wrong patient population
Mmasa 2018	Gestational diabetes in women on dolutegravir- or efavirenz-based ART in Botswana	oral abstract
Mnabwiru 2024	mpact of advanced maternal age on perinatal outcomes in Tanzania: Insights from Kilimanjaro Christian Medical Center Birth Registry.	Wrong study design
Mohammed 2018	Correlation between circulating level of tumor necrosis factor-alpha and insulin resistance in Nigerian women with gestational diabetes mellitus.	Wrong study design
Mohammed 2018	Maternal serum level of TNF-alpha in Nigerian women with gestational diabetes mellitus.	Wrong study design

Msollo 2019	Insulin Resistance Among Pregnant Women in Urban Areas of Arusha Region, Tanzania.	Subset or repeat of included study
Msollo 2022	Simple method for identification of women at risk of gestational diabetes mellitus in Arusha urban, Tanzania.	Wrong study design
Muche 2019	Prevalence and determinants of gestational diabetes mellitus in Africa based on the updated international diagnostic criteria: A systematic review and meta-analysis	systematic review
Muche 2020	Gestational diabetes mellitus increased the risk of adverse neonatal outcomes: A prospective cohort study in Northwest Ethiopia	Subset or repeat of included study
Muche 2020	Effects of gestational diabetes mellitus on risk of adverse maternal outcomes: a prospective cohort study in Northwest Ethiopia.	Subset or repeat of included study
Mukuve 2020	Magnitude of screening for gestational diabetes mellitus in an urban setting in Tanzania; a cross-sectional analytic study.	Wrong study design
Munang 2017	Reproducibility of the 75 g oral glucose tolerance test for the diagnosis of gestational diabetes mellitus in a sub-Saharan African population.	Wrong study design
Musa 2024	Kisspeptin signalling and its correlation with placental ultrastructure and clinical outcomes in pregnant South African women with obesity and gestational diabetes.	no prevalence cited
Mutabazi 2021	Integrating gestational diabetes and type 2 diabetes care into primary health care: Lessons from prevention of mother-to-child transmission of HIV in South Africa - A mixed methods study.	no prevalence cited
MUTABAZI 2022	Integrating Gestational Diabetes Screening and Care and Type 2 Diabetes Mellitus Prevention After GDM Into Community Based Primary Health Care in South Africa-Mixed Method Study.	Wrong study design
Muyer 2008	Diabetes mellitus in Sub-Saharan Africa: A systematic review of the literature	systematic review
Mwanri 2015	Gestational diabetes mellitus in sub-Saharan Africa: systematic review and metaregression on prevalence and risk factors.	systematic review
Nabwera 2019	Burden of disease in neonatal units in Nigeria and Kenya	oral abstract
Nakanga 2022	The contribution of maternal glucose to birth weight is less in Uganda (sub-Saharan Africa) compared to white and black ethnic groups in the HAPO study	oral abstract
Nakanga 2024	The Contribution of Maternal Glucose to Birth Weight is Smaller in Uganda (Sub-Saharan Africa) than in Afro-Caribbean or White Ethnicity Mother-Child Pairs from Outside Africa	Wrong study design
Napoli 2015	Prevalent gestational diabetes mellitus in HIV-infected and-uninfected pregnant women living in sub-Saharan Africa	poster
Natamba 2019	Burden, risk factors and maternal and offspring outcomes of gestational diabetes mellitus (GDM) in sub-Saharan Africa (SSA): a systematic review and meta-analysis.	systematic review
Nhidza 2018	Diagnosis of gestational diabetes mellitus in Urban Harare, Zimbabwe	own diagnostic criteria
Nicolaou 2020	Maternal and neonatal outcomes following the introduction of oral hypoglycaemic agents for gestational diabetes mellitus were comparable to insulin monotherapy in two historical cohorts.	Wrong study design
Nicolaou 2021	The metabolic outcomes following hyperglycaemia first detected in pregnancy in Soweto, South Africa	reference incorrect
Nicolaou 2022	Perspectives on gestational diabetes mellitus in South Africa.	systematic review
Nisar 2024	Early to mid-pregnancy HbA1c levels and its association with adverse pregnancy outcomes in three low middle-income countries in Asia and Sub-Saharan Africa.	Wrong study design
Niyibizi 2016	Gestational Diabetes Mellitus and Its Associated Risk Factors in Pregnant Women at Selected Health Facilities in Kigali City, Rwanda	fasting blood glucose only
Njete 2017	Prevalence, predictors and challenges of gestational diabetes mellitus screening among pregnant women in northern Tanzania	Subset or repeat of included study
Nombo 2018	Gestational diabetes mellitus risk score: A practical tool to predict gestational diabetes mellitus risk in Tanzania.	Subset or repeat of included study
Norris 2017	The co-existence of HIV infection and obesity with risk of gestational diabetes mellitus in black Africans in Sub Saharan Africa	oral abstract
Ntshauba 2022	Prevalence and Associated Risk Factors of Gestational Diabetes Mellitus in Limpopo Province, South Africa.	no diagnostic criteria specified
Nwaokoro 2013	Risk Factors Associated with Gestational Diabetes among Pregnant Women in Owerri Municipal Council, Southeastern Nigeria	Wrong study design
O 2013	Prevalence Of Gestational Diabetes Mellitus; Risk Factors Among Pregnant Women (In Abakaliki Metropolis, Ebonyi State Nigeria.)	own diagnostic criteria
Oboro 2006	Pregnancy outcome in nulliparous women aged 35 or older.	no diagnostic criteria specified
Odar 2004	Maternal and fetal outcome of gestational diabetes mellitus in Mulago Hospital, Uganda	Wrong study design
Oderinde 2013	Isolation of enterovirus from feacal samples of patients with diabetes mellitus in Maiduguri, Nigeria	Wrong patient population
Ogbera 2014	Diabetes mellitus in Nigeria: The past, present and future	systematic review
Ogu 2017	Assessing the outcome of universal vs. Risk-based screening for GDM in resource limited setting of the niger-delta	oral abstract
Ogu 2017	Screening for Gestational Diabetes Mellitus: Findings from a Resource Limited Setting of Nigeria	Wrong study design
Ogu 2022	The Case for Early and Universal Screening for Gestational Diabetes Mellitus: Findings from 9314 Pregnant Women in a Major City in Nigeria.	fasting blood glucose only

Okonofua 1988	Criteria for the oral glucose tolerance test in pregnant and non-pregnant Nigerian women.	prior to 1990
Okonofua 1995	An evaluation of the WHO criteria for abnormal glucose tolerance test during pregnancy in Nigerian women.	no prevalence cited
Okunowo 2019	Role of risk factors for gestational diabetes mellitus in determining newborn outcomes in a Nigerian teaching hospital	poster
Okunowo 2020	Screening for Gestational Diabetes Mellitus: Universal or Selective Screening? Screening for Gestational Diabetes Mellitus: Universal or Selective Screening?	oral abstract
OkunowoBolanle 2023	Screening for gestational diabetes mellitus: universal or selective screening?	oral abstract
Olagbuji 2022	A multicenter prospective study of early gestational diabetes mellitus: Rates, severity, and risk factors based on IADPSG-defined fasting glycemia.	Wrong study design
Olarinoye 2004	Diagnosis of gestational diabetes mellitus in Nigerian pregnant women--comparison between 75G and 100G oral glucose tolerance tests.	Wrong study design
Olumodeji 2020	Implementing the 2013 WHO diagnostic criteria for gestational diabetes mellitus in a Rural Nigerian Population.	own diagnostic criteria
Om 2017	Risk Factors of Gestational Diabetes Mellitus in a Reference Maternal Health Care Centre in Southern Benin	Wrong study design
Opara 2010	Morbidity and mortality amongst infants of diabetic mothers admitted into a special care baby unit in Port Harcourt, Nigeria.	Wrong patient population
Oppong 2020	Risk factors and pregnancy outcome in women aged over 40 years at Korle-Bu Teaching Hospital in Accra, Ghana.	no diagnostic criteria specified
Oputa 2013	Gestational diabetes Mellitus: A clinical challenge in Africa	systematic review
Orru 2017	Screening for gestational diabetes: evaluation of prevalence in age-stratified subgroups at Central hospital Warri Nigeria	Wrong study design
Otim 1974	Diabetes and pregnancy. Problems, shortcomings and future possibilities of management at Mulago Hospital.	prior to 1990
Otolorin 1985	Reproductive performance following active management of diabetic pregnancies at the University College Hospital, Ibadan, Nigeria.	prior to 1990
Parkhi 2023	Systematic Review of risk score prediction models using maternal characteristics with and without biomarkers for the prediction of GDM	systematic review
Pastakia 2021	Risk of Dysglycemia in Pregnancy amongst Kenyan Women with HIV Infection: A Nested Case-Control Analysis from the STRiDE Study.	Subset or repeat of included study
Pheiffer 2018	Decreased Expression of Circulating miR-20a-5p in South African Women with Gestational Diabetes Mellitus.	Wrong study design
Pheiffer 2021	Altered epigenetic signatures in South African women with gestational diabetes mellitus	oral abstract
Pheiffer 2021	Altered epigenetic signatures in South African women with gestational diabetes mellitus	Wrong study design
Putoto 2020	A simplified diagnostic work-up for the detection of gestational diabetes mellitus in low resources settings: achievements and challenges.	own diagnostic criteria
Ramtoola 2001	estational impaired glucose tolerance does not increase perinatal mortality in a developing country: cohort study.	Wrong study design
Ranchod 1991	Incidence of gestational diabetes at Northdale Hospital, Pietermaritzburg.	prior to 1990
Rayis 2011	Obesity and pregnancy outcome in Khartoum, Sudan	no diagnostic criteria specified
Rayis 2020	Reliability of glycosylated hemoglobin in the diagnosis of gestational diabetes mellitus.	Subset or repeat of included study
Rossouw 2017	An audit of stillborn babies in mothers with diabetes mellitus at a tertiary South African Hospital	Wrong study design
Sagna 2017	Prevalence, associated factors and evolution of gestational diabetes mellitus in Ouagadougou	poster
Sagna 2023	Prevalence of gestational diabetes mellitus and factors associated in two Burkina Faso urban hospitals	oral abstract
Seifu 2024	Association of overweight and obesity with gestational diabetes mellitus among pregnant women attending antenatal care clinics in Addis Ababa, Ethiopia: a case-control study.	Wrong study design
Sekitoleko 2022	The influence of fasting and post-load glucose levels on maternal and neonatal outcomes in women with hyperglycaemia in pregnancy in Uganda: A prospective observational cohort study.	Subset or repeat of included study
Senbanjo 2021	Early pregnancy body mass index, gestational weight gain and perinatal outcome in an obstetric population in Lagos, Nigeria.	no prevalence cited
Sewor 2024	Fruits and vegetables intake improves birth outcomes of women with gestational diabetes mellitus and hypertensive disorders of pregnancy.	Wrong study design
Sidibe 1994	Diabetic pregnancy in Senegal (years 1980 to 1989)].	prior to 1990
Sidibe 1996	Diabetic pregnancy in Black Africans	reference incorrect
Soepnel 2019	Maternal and neonatal outcomes following a diabetic pregnancy within the context of HIV.	Wrong patient population
Sonuga 2020	Hypovitaminosis D Is Associated with Some Metabolic Indices in Gestational Diabetes Mellitus.	Wrong study design
Swai 1991	No deterioration of oral glucose tolerance during pregnancy in rural Tanzania.	Wrong study design

Tandu-Umba 2012	Outcome-based diagnosis of hyperglycemia in pregnancy in Kinshasa, Democratic Republic of Congo	fasting blood glucose only
Tandu-Umba 2013	Outcome-based diagnosis of hyperglycemia in pregnancy in Kinshasa, Democratic Republic of Congo.	Subset or repeat of included study
Taye 2022	Previous adverse pregnancy events as a predictor of gestational diabetes mellitus in Southern Ethiopia: a case control study.	Wrong study design
Tesfaye 2023	Determinants of puerperal sepsis among postpartum women at public hospitals of Hawassa city, Southern Ethiopia: Institution-based unmatched case-control study.	Wrong study design
Tewabe 2024	Gestational diabetes mellitus and its association with ABO blood group type among pregnant women with pregnancy-induced hypertension in Northwest Ethiopia: A comparative study	Wrong patient population
Tibebu 2023	Prevalence of birth injuries and associated factors among newborns delivered in public hospitals Addis Ababa, Ethiopia, 2021. Crossectional study.	no diagnostic criteria specified
Tolefac 2017	Ten years analysis of stillbirth in a tertiary hospital in sub-Sahara Africa: a case control study.	no diagnostic criteria specified
Ugboma 2014	Gestational Diabetes: Risk Factors, Perinatal Complications and Screening Importance in Niger Delta Region of Nigeria: A Public Health Dilemma	no diagnostic criteria specified
Umez-Eronini 1978	Serial oral glucose tolerance studies in primigravid Nigerian women.	prior to 1990
vanBogaert 1998	Gestational diabetes mellitus--are African diagnostic criteria warranted?	oral abstract
VanZyl 2018	Pregnancy outcome in patients with pregestational and gestational diabetes attending Groote Schuur Hospital, Cape Town, South Africa.	Wrong study design
Vogel 2014	Maternal morbidity and preterm birth in 22 low- and middle-income countries: A secondary analysis of the WHO Global Survey dataset	no prevalence cited
Wakwoya 2018	Adverse maternal outcome and its association with gestational diabetes among women who gave birth in selected public hospitals in Eastern Ethiopia	Wrong study design
Wang 2022	IDF Diabetes Atlas: Estimation of Global and Regional Gestational Diabetes Mellitus Prevalence for 2021 by International Association of Diabetes in Pregnancy Study Group's Criteria	systematic review
Wokoma 2001	Gestational Diabetes Mellitus in a Nigerian Antenatal Population	own diagnostic criteria
Wondemagegn 2017	Undiagnosed Diabetes Mellitus and Related Factors in East Gojjam (NW Ethiopia) in 2016: A Community-Based Study.	Wrong patient population
Zar 2019	Maternal health and birth outcomes in a South African birth cohort study.	Wrong study design
Zeck 2009	Gestational diabetes in East Africa: a mostly disregarded disease?	Wrong study design

Text S2 – overall and subgroup meta-analysis results

```
ies.da <- escalc(xi=cases, ni = size, data = rtest, measure = "PFT")
> # pool the derived effect sizes using random effects model
> pes.da <- rma(yi, vi, data = ies.da, method = "REML")
> summary(pes.da)
Random-Effects Model (k = 49; tau^2 estimator: REML)
  logLik  deviance       AIC       BIC      AICc
 31.6451  -63.2901  -59.2901  -55.5477  -59.0235

tau^2 (estimated amount of total heterogeneity): 0.0149 (SE = 0.0032)
tau (square root of estimated tau^2 value):      0.1221
I^2 (total heterogeneity / total variability):   97.05%
H^2 (total variability / sampling variability):  33.92

Test for Heterogeneity:
Q(df = 48) = 1419.2239, p-val < .0001

Model Results:
estimate      se     zval    pval   ci.lb   ci.ub
  0.3846  0.0178  21.5579  <.0001  0.3497  0.4196  ***
---
Signif. codes:  0 ‘***’ 0.001 ‘**’ 0.01 ‘*’ 0.05 ‘.’ 0.1 ‘ ’ 1


#Inverse of double arcsine transformation to get back to proportions
> pes <- predict(pes.da, transf = transf.ipft.hm, targ=list(ni=rtest$size))
> print(pes)
   pred  ci.lb  ci.ub  pi.lb  pi.ub
 0.1398 0.1163 0.1650 0.0189 0.3432


#heterogeneity analysis
> print(pes.da, digits = 4)
Random-Effects Model (k = 49; tau^2 estimator: REML)
tau^2 (estimated amount of total heterogeneity): 0.0149 (SE = 0.0032)
tau (square root of estimated tau^2 value):      0.1221
I^2 (total heterogeneity / total variability):   97.05%
H^2 (total variability / sampling variability):  33.92

Test for Heterogeneity:
Q(df = 48) = 1419.2239, p-val < .0001

Model Results:

estimate      se     zval    pval   ci.lb   ci.ub
  0.3846  0.0178  21.5579  <.0001  0.3497  0.4196  ***

Signif. codes:  0 ‘***’ 0.001 ‘**’ 0.01 ‘*’ 0.05 ‘.’ 0.1 ‘ ’ 1

> confint(pes.da, digits = 4)
       estimate   ci.lb   ci.ub
tau^2    0.0149  0.0102  0.0238
tau      0.1221  0.1008  0.1542
I^2(%)  97.0521 95.7394 98.1322
H^2     33.9226 23.4707 53.5385
```

```
pes.country <- metaprop(cases, size, label, data=rtest, sm="PFT", method.tau = "DL"
, method.ci = "NAsm", subgroup= country)
> summary(pes.country)
                         proportion           95%-CI %W(common) %W(random)      country
Khambule 2025               0.0771 [0.0619; 0.0939]        3.9        2.1 South Africa
Basil 2023                  0.1673 [0.1258; 0.2133]        1.0        2.0      Nigeria
Bruno 2024                  0.1765 [0.1357; 0.2213]        1.1        2.0      Nigeria
Kahimakazi 2023             0.1020 [0.0721; 0.1365]        1.2        2.0       Uganda
Bengtson 2023               0.0605 [0.0389; 0.0862]        1.4        2.1 South Africa
Agbozo 2021                 0.2646 [0.2246; 0.3066]        1.6        2.1        Ghana
Amadi 2021                  0.1440 [0.1030; 0.1904]        0.9        2.0      Nigeria
Abindu 2024                 0.0745 [0.0408; 0.1169]        0.7        2.0       Uganda
Sobngwi 2023                0.1759 [0.1522; 0.2010]        3.4        2.1     Cameroon
Alassani2022                0.1258 [0.0772; 0.1839]        0.5        1.9        Benin
Akinyemi 2023               0.0778 [0.0485; 0.1130]        1.0        2.0      Nigeria
Nigatu 2022                 0.1692 [0.1336; 0.2082]        1.4        2.0     Ethiopia
Mdoe 2021                   0.2749 [0.2394; 0.3120]        2.1        2.1     Tanzania
Nwali 2021                  0.1151 [0.0852; 0.1487]        1.4        2.0      Nigeria
Milln 2021                  0.0812 [0.0716; 0.0914]       10.6        2.1       Uganda
Mmasa 2021                  0.0844 [0.0612; 0.1108]        1.8        2.1     Botswana
Rayis 2021                  0.1853 [0.1402; 0.2351]        0.9        2.0        Sudan
Prioreschi 2021             0.1147 [0.0939; 0.1374]        3.0        2.1 South Africa
Mghanga 2020                0.0425 [0.0278; 0.0600]        2.2        2.1     Tanzania
Maidwell-Smith 2020         0.1457 [0.0998; 0.1984]        0.7        2.0   The Gambia
Meharry 2019                0.0320 [0.0141; 0.0563]        1.0        2.0       Rwanda
Muche 2019                  0.1276 [0.1078; 0.1487]        3.7        2.1     Ethiopia
Msollo 2019                 0.1303 [0.1013; 0.1624]        1.7        2.1     Tanzania
Naser 2019                  0.1508 [0.0931; 0.2191]        0.5        1.9        Sudan
Egbe 2018                   0.2050 [0.1517; 0.2639]        0.7        2.0     Cameroon
Macaulay 2018               0.0913 [0.0788; 0.1046]        6.9        2.1 South Africa
Njete 2018                  0.1952 [0.1543; 0.2396]        1.2        2.0     Tanzania
Olagbuji 2017               0.1571 [0.1167; 0.2023]        1.0        2.0      Nigeria
Pastakia 2017               0.0292 [0.0172; 0.0442]        2.2        2.1        Kenya
Nakabuye 2017               0.3028 [0.2474; 0.3612]        0.9        2.0       Uganda
Adam 2017                   0.2581 [0.2225; 0.2954]        2.0        2.1 South Africa
Oppong 2015                 0.0927 [0.0661; 0.1233]        1.4        2.1        Ghana
Olagbuji 2015               0.0859 [0.0698; 0.1036]        3.8        2.1      Nigeria
Minsart 2014                0.4589 [0.3949; 0.5235]        0.8        2.0     Djibouti
Mwanri 2014                 0.1322 [0.1108; 0.1552]        3.3        2.1     Tanzania
Senbanjo 2023               0.1900 [0.1530; 0.2300]        1.5        2.1      Nigeria
Bune 2024                   0.1606 [0.1340; 0.1891]        2.5        2.1     Ethiopia
Saravanan 2024              0.0303 [0.0235; 0.0381]        7.8        2.1        Kenya
Dickson 2020                0.0696 [0.0504; 0.0917]        2.1        2.1 South Africa
Grunnett 2020               0.3903 [0.3425; 0.4392]        1.4        2.0     Tanzania
Momo 2021                   0.1679 [0.1377; 0.2004]        2.0        2.1       Guinea
Jibrin 2020                 0.1319 [0.0951; 0.1737]        1.0        2.0      Nigeria
Inaku 2021                  0.1391 [0.1045; 0.1778]        1.3        2.0      Nigeria
Onyenekwe 2019              0.3592 [0.2820; 0.4401]        0.5        1.9      Nigeria
Oriji 2017                  0.1489 [0.1061; 0.1975]        0.9        2.0      Nigeria
Woticha 2018                0.0425 [0.0266; 0.0617]        1.9        2.1     Ethiopia
Phiri 2021                  0.2487 [0.1901; 0.3123]        0.7        2.0       Malawi
Boda 2021                   0.0711 [0.0472; 0.0992]        1.4        2.0     Ethiopia
OumouHawaBah 2022           0.1388 [0.1148; 0.1647]        2.7        2.1       Guinea

Number of studies: k = 49
Number of observations: o = 27540
Number of events: e = 3406
```

```
                     proportion           95%-CI
Common effect model      0.1140 [0.1102; 0.1178]
Random effects model     0.1396 [0.1176; 0.1632]

Quantifying heterogeneity (with 95%-CIs):
 tau^2 = 0.0129 [0.0094; 0.0230]; tau = 0.1137 [0.0972; 0.1518]
 I^2 = 96.6% [96.1%; 97.1%]; H = 5.44 [5.04; 5.87]

Test of heterogeneity:
       Q d.f.  p-value
 1419.22   48 < 0.0001

Results for subgroups (common effect model):
                         k proportion           95%-CI      Q   I^2
country = Benin          1     0.1258 [0.0772; 0.1839]   0.00    --
country = Botswana       1     0.0844 [0.0612; 0.1108]   0.00    --
country = Cameroon       2     0.1805 [0.1586; 0.2034]   0.98  0.0%
country = Djibouti       1     0.4589 [0.3949; 0.5235]   0.00    --
country = Ethiopia       5     0.1139 [0.1027; 0.1256]  70.52 94.3%
country = Ghana          2     0.1748 [0.1499; 0.2013]  44.49 97.8%
country = Guinea         2     0.1509 [0.1318; 0.1710]   2.07 51.6%
country = Kenya          2     0.0299 [0.0239; 0.0367]   0.01  0.0%
country = Malawi         1     0.2487 [0.1901; 0.3123]   0.00    --
country = Nigeria       12     0.1339 [0.1238; 0.1444]  94.61 88.4%
country = Rwanda         1     0.0320 [0.0141; 0.0563]   0.00    --
country = South Africa   6     0.1003 [0.0924; 0.1085] 124.39 96.0%
country = Sudan          2     0.1735 [0.1371; 0.2133]   0.66  0.0%
country = Tanzania       6     0.1646 [0.1520; 0.1775] 266.20 98.1%
country = The Gambia     1     0.1457 [0.0998; 0.1984]   0.00    --
country = Uganda         4     0.0932 [0.0839; 0.1028]  81.23 96.3%

Test for subgroup differences (common effect model):
                    Q d.f.  p-value
Between groups 734.06   15 < 0.0001
Within groups  685.16   33 < 0.0001

Results for subgroups (random effects model):
                         k proportion           95%-CI  tau^2    tau
country = Benin          1     0.1258 [0.0772; 0.1839]     --     --
country = Botswana       1     0.0844 [0.0612; 0.1108]     --     --
country = Cameroon       2     0.1805 [0.1586; 0.2034]      0      0
country = Djibouti       1     0.4589 [0.3949; 0.5235]     --     --
country = Ethiopia       5     0.1089 [0.0656; 0.1614] 0.0072 0.0849
country = Ghana          2     0.1701 [0.0388; 0.3671] 0.0258 0.1606
country = Guinea         2     0.1520 [0.1246; 0.1816] 0.0004 0.0206
country = Kenya          2     0.0299 [0.0239; 0.0367]      0      0
country = Malawi         1     0.2487 [0.1901; 0.3123]     --     --
country = Nigeria       12     0.1505 [0.1194; 0.1845] 0.0056 0.0746
country = Rwanda         1     0.0320 [0.0141; 0.0563]     --     --
country = South Africa   6     0.1052 [0.0665; 0.1516] 0.0071 0.0845
country = Sudan          2     0.1735 [0.1371; 0.2133]      0      0
country = Tanzania       6     0.1803 [0.0941; 0.2868] 0.0244 0.1561
country = The Gambia     1     0.1457 [0.0998; 0.1984]     --     --
country = Uganda         4     0.1290 [0.0588; 0.2211] 0.0146 0.1207

Test for subgroup differences (random effects model):
                    Q d.f.  p-value
Between groups 575.73   15 < 0.0001
```

```
Details of meta-analysis methods:
- Inverse variance method
- DerSimonian-Laird estimator for tau^2
- Jackson method for confidence interval of tau^2 and tau
- Calculation of I^2 based on Q
- Freeman-Tukey double arcsine transformation
- Normal approximation confidence interval for individual studies
```

```
pes.subregion <- metaprop(cases, size, label, data=rtest, sm="PFT", method.tau = "D
L", method.ci = "NAsm", subgroup= subregion)
> summary(pes.subregion)
                    proportion           95%-CI %W(common) %W(random) subregion
Khambule 2025           0.0771 [0.0619; 0.0939]        3.9        2.1     south
Basil 2023              0.1673 [0.1258; 0.2133]        1.0        2.0      west
Bruno 2024              0.1765 [0.1357; 0.2213]        1.1        2.0      west
Kahimakazi 2023         0.1020 [0.0721; 0.1365]        1.2        2.0      east
Bengtson 2023           0.0605 [0.0389; 0.0862]        1.4        2.1     south
Agbozo 2021             0.2646 [0.2246; 0.3066]        1.6        2.1      west
Amadi 2021              0.1440 [0.1030; 0.1904]        0.9        2.0      west
Abindu 2024             0.0745 [0.0408; 0.1169]        0.7        2.0      east
Sobngwi 2023            0.1759 [0.1522; 0.2010]        3.4        2.1   central
Adebayo 2022            0.1258 [0.0772; 0.1839]        0.5        1.9      west
Akinyemi 2023           0.0778 [0.0485; 0.1130]        1.0        2.0      west
Nigatu 2022             0.1692 [0.1336; 0.2082]        1.4        2.0      east
Mdoe 2021               0.2749 [0.2394; 0.3120]        2.1        2.1      east
Nwali 2021              0.1151 [0.0852; 0.1487]        1.4        2.0      west
Milln 2021              0.0812 [0.0716; 0.0914]       10.6        2.1      east
Mmasa 2021              0.0844 [0.0612; 0.1108]        1.8        2.1     south
Rayis 2021              0.1853 [0.1402; 0.2351]        0.9        2.0      east
Prioreschi 2021         0.1147 [0.0939; 0.1374]        3.0        2.1     south
Mghanga 2020            0.0425 [0.0278; 0.0600]        2.2        2.1      east
Maidwell-Smith 2020     0.1457 [0.0998; 0.1984]        0.7        2.0      west
Meharry 2019            0.0320 [0.0141; 0.0563]        1.0        2.0      east
Muche 2019              0.1276 [0.1078; 0.1487]        3.7        2.1      east
Msollo 2019             0.1303 [0.1013; 0.1624]        1.7        2.1      east
Naser 2019              0.1508 [0.0931; 0.2191]        0.5        1.9      east
Egbe 2018               0.2050 [0.1517; 0.2639]        0.7        2.0   central
Macaulay 2018           0.0913 [0.0788; 0.1046]        6.9        2.1     south
Njete 2018              0.1952 [0.1543; 0.2396]        1.2        2.0      east
Olagbuji 2017           0.1571 [0.1167; 0.2023]        1.0        2.0      west
Pastakia 2017           0.0292 [0.0172; 0.0442]        2.2        2.1      east
Nakabuye 2017           0.3028 [0.2474; 0.3612]        0.9        2.0      east
Adam 2017               0.2581 [0.2225; 0.2954]        2.0        2.1     south
Oppong 2015             0.0927 [0.0661; 0.1233]        1.4        2.1      west
Olagbuji 2015           0.0859 [0.0698; 0.1036]        3.8        2.1      west
Minsart 2014            0.4589 [0.3949; 0.5235]        0.8        2.0      east
Mwanri 2014             0.1322 [0.1108; 0.1552]        3.3        2.1      east
Senbanjo 2023           0.1900 [0.1530; 0.2300]        1.5        2.1      west
Bune 2024               0.1606 [0.1340; 0.1891]        2.5        2.1      east
Saravanan 2024          0.0303 [0.0235; 0.0381]        7.8        2.1      east
Dickson 2020            0.0696 [0.0504; 0.0917]        2.1        2.1     south
Grunnett 2020           0.3903 [0.3425; 0.4392]        1.4        2.0      east
Momo 2021               0.1679 [0.1377; 0.2004]        2.0        2.1      west
Jibrin 2020             0.1319 [0.0951; 0.1737]        1.0        2.0      west
Inaku 2021              0.1391 [0.1045; 0.1778]        1.3        2.0      west
Onyenekwe 2019          0.3592 [0.2820; 0.4401]        0.5        1.9      west
```

```
Oriji 2017            0.1489 [0.1061; 0.1975]        0.9        2.0      west
Wolka 2018            0.0425 [0.0266; 0.0617]        1.9        2.1      east
PhiriTamara 2021      0.2487 [0.1901; 0.3123]        0.7        2.0      east
Boda 2021             0.0711 [0.0472; 0.0992]        1.4        2.0      east
OumouHawaBah 2022     0.1388 [0.1148; 0.1647]        2.7        2.1      west

Number of studies: k = 49
Number of observations: o = 27540
Number of events: e = 3406

                     proportion           95%-CI
Common effect model      0.1140 [0.1102; 0.1178]
Random effects model     0.1396 [0.1176; 0.1632]

Quantifying heterogeneity (with 95%-CIs):
 tau^2 = 0.0129 [0.0094; 0.0230]; tau = 0.1137 [0.0972; 0.1518]
 I^2 = 96.6% [96.1%; 97.1%]; H = 5.44 [5.04; 5.87]

Test of heterogeneity:
       Q d.f.  p-value
 1419.22   48 < 0.0001

Results for subgroups (common effect model):
                      k proportion           95%-CI       Q   I^2
subregion = south     7     0.0989 [0.0914; 0.1067]  125.69 95.2%
subregion = west     18     0.1421 [0.1338; 0.1506]  151.11 88.7%
subregion = east     22     0.1029 [0.0979; 0.1081] 1021.22 97.9%
subregion = central   2     0.1805 [0.1586; 0.2034]    0.98  0.0%

Test for subgroup differences (common effect model):
                     Q d.f.  p-value
Between groups  120.22    3 < 0.0001
Within groups  1299.00   45 < 0.0001

Results for subgroups (random effects model):
                      k proportion           95%-CI  tau^2    tau
subregion = south     7     0.1022 [0.0683; 0.1420] 0.0063 0.0796
subregion = west     18     0.1515 [0.1262; 0.1787] 0.0054 0.0733
subregion = east     22     0.1389 [0.0998; 0.1831] 0.0199 0.1412
subregion = central   2     0.1805 [0.1586; 0.2034]      0      0

Test for subgroup differences (random effects model):
                   Q d.f. p-value
Between groups 12.36    3  0.0062

Details of meta-analysis methods:
- Inverse variance method
- DerSimonian-Laird estimator for tau^2
- Jackson method for confidence interval of tau^2 and tau
- Calculation of I^2 based on Q
- Freeman-Tukey double arcsine transformation
- Normal approximation confidence interval for individual studies
```

```
> metareg.moderators <- rma(yi, vi, data = ies.da, mods = ~ study_size + lab_test +
sample_frame + criteria + study_age + quality)
> summary(metareg.moderators)

Mixed-Effects Model (k = 49; tau^2 estimator: REML)

  logLik  deviance       AIC       BIC      AICc
 27.9956  -55.9911  -29.9911   -9.0492  -14.1650

tau^2 (estimated amount of residual heterogeneity):     0.0122 (SE = 0.0030)
tau (square root of estimated tau^2 value):             0.1106
I^2 (residual heterogeneity / unaccounted variability): 95.97%
H^2 (unaccounted variability / sampling variability):   24.80
R^2 (amount of heterogeneity accounted for):            17.85%

Test for Residual Heterogeneity:
QE(df = 37) = 869.2037, p-val < .0001

Test of Moderators (coefficients 2:12):
QM(df = 11) = 21.1855, p-val = 0.0315

Model Results:

                             estimate      se     zval    pval    ci.lb   ci.ub
intrcpt                        0.2608  0.0641   4.0720  <.0001   0.1353  0.3864  ***
study_size0-299                0.1193  0.0471   2.5313  0.0114   0.0269  0.2117    *
study_size300-699              0.0628  0.0473   1.3274  0.1844  -0.0299  0.1556
lab_testpoc                    0.1078  0.0422   2.5530  0.0107   0.0250  0.1906    *
lab_testunknown               -0.0007  0.0820  -0.0086  0.9931  -0.1614  0.1600
sample_frameLocal             -0.0249  0.0490  -0.5079  0.6115  -0.1209  0.0712
sample_framemulti-city/reg    -0.0171  0.0470  -0.3638  0.7160  -0.1092  0.0750
criteriaIADPSGm               -0.1010  0.0635  -1.5910  0.1116  -0.2255  0.0234
criteriaWHO2013               -0.0298  0.0427  -0.6994  0.4843  -0.1135  0.0538
study_ageold                   0.0341  0.0524   0.6508  0.5152  -0.0687  0.1369
study_ageolder                 0.1455  0.0692   2.1010  0.0356   0.0098  0.2812    *
qualitymedium/low              0.0569  0.0576   0.9882  0.3231  -0.0560  0.1698

---
Signif. codes:  0 ‘***’ 0.001 ‘**’ 0.01 ‘*’ 0.05 ‘.’ 0.1 ‘ ’ 1
```

Figure S1 – Overall and country subgroup forest plots

Study	Events	Total	Weight	IV, Random, 95% CI
Milln 2021	237	2917	2.1%	0.08 [0.07; 0.09]
Saravanan 2024	65	2142	2.1%	0.03 [0.02; 0.04]
Macaulay 2018	174	1906	2.1%	0.09 [0.08; 0.10]
Khambule 2025	83	1076	2.1%	0.08 [0.06; 0.09]
Olagbuji 2015	91	1059	2.1%	0.09 [0.07; 0.10]
Muche 2019	131	1027	2.1%	0.13 [0.11; 0.15]
Sobngwi 2023	165	938	2.1%	0.18 [0.15; 0.20]
Mwanri 2014	119	900	2.1%	0.13 [0.11; 0.15]
Prioreschi 2021	95	828	2.1%	0.11 [0.09; 0.14]
OumouHawaBah 2022	103	742	2.1%	0.14 [0.11; 0.16]
Bune 2024	110	685	2.1%	0.16 [0.13; 0.19]
Pastakia 2017	18	616	2.1%	0.03 [0.02; 0.04]
Mghanga 2020	26	612	2.1%	0.04 [0.03; 0.06]
Dickson 2020	41	589	2.1%	0.07 [0.05; 0.09]
Mdoe 2021	160	582	2.1%	0.27 [0.24; 0.31]
Adam 2017	143	554	2.1%	0.26 [0.22; 0.30]
Momo 2021	92	548	2.1%	0.17 [0.14; 0.20]
Woticha 2018	22	518	2.1%	0.04 [0.03; 0.06]
Mmasa 2021	41	486	2.1%	0.08 [0.06; 0.11]
Msollo 2019	61	468	2.1%	0.13 [0.10; 0.16]
Agbozo 2021	118	446	2.1%	0.26 [0.22; 0.31]
Senbanjo 2023	76	400	2.1%	0.19 [0.15; 0.23]
Oppong 2015	37	399	2.1%	0.09 [0.07; 0.12]
Bengtson 2023	24	397	2.1%	0.06 [0.04; 0.08]
Grunnett 2020	153	392	2.0%	0.39 [0.34; 0.44]
Nwali 2021	45	391	2.0%	0.12 [0.09; 0.15]
Nigatu 2022	66	390	2.0%	0.17 [0.13; 0.21]
Boda 2021	27	380	2.0%	0.07 [0.05; 0.10]
Inaku 2021	48	345	2.0%	0.14 [0.10; 0.18]
Kahimakazi 2023	35	343	2.0%	0.10 [0.07; 0.14]
Njete 2018	65	333	2.0%	0.20 [0.15; 0.24]
Bruno 2024	54	306	2.0%	0.18 [0.14; 0.22]
Jibrin 2020	38	288	2.0%	0.13 [0.10; 0.17]
Basil 2023	47	281	2.0%	0.17 [0.13; 0.21]
Meharry 2019	9	281	2.0%	0.03 [0.02; 0.06]
Olagbuji 2017	44	280	2.0%	0.16 [0.12; 0.20]
Akinyemi 2023	21	270	2.0%	0.08 [0.05; 0.11]
Rayis 2021	48	259	2.0%	0.19 [0.14; 0.24]
Nakabuye 2017	76	251	2.0%	0.30 [0.25; 0.36]
Amadi 2021	36	250	2.0%	0.14 [0.10; 0.19]
Oriji 2017	35	235	2.0%	0.15 [0.11; 0.20]
Minsart 2014	106	231	2.0%	0.46 [0.40; 0.52]
Egbe 2018	41	200	2.0%	0.20 [0.15; 0.26]
Maidwell–Smith 2020	29	199	2.0%	0.15 [0.10; 0.20]
Phiri 2021	48	193	2.0%	0.25 [0.19; 0.31]
Abindu 2024	14	188	2.0%	0.07 [0.04; 0.12]
Alassani 2022	19	151	1.9%	0.13 [0.08; 0.18]
Onyenekwe 2019	51	142	1.9%	0.36 [0.28; 0.44]
Naser 2019	19	126	1.9%	0.15 [0.10; 0.22]
**Total (95% CI)**		27540	100.0%	**0.14 [0.12; 0.16]**
**Prediction interval**				**[0.02; 0.33]**

Heterogeneity: $Tau^2 = 0.0129$; $Chi^2 = 1419.22$, df = 48 ($P < 0.0001$); $I^2 = 96.6\%$

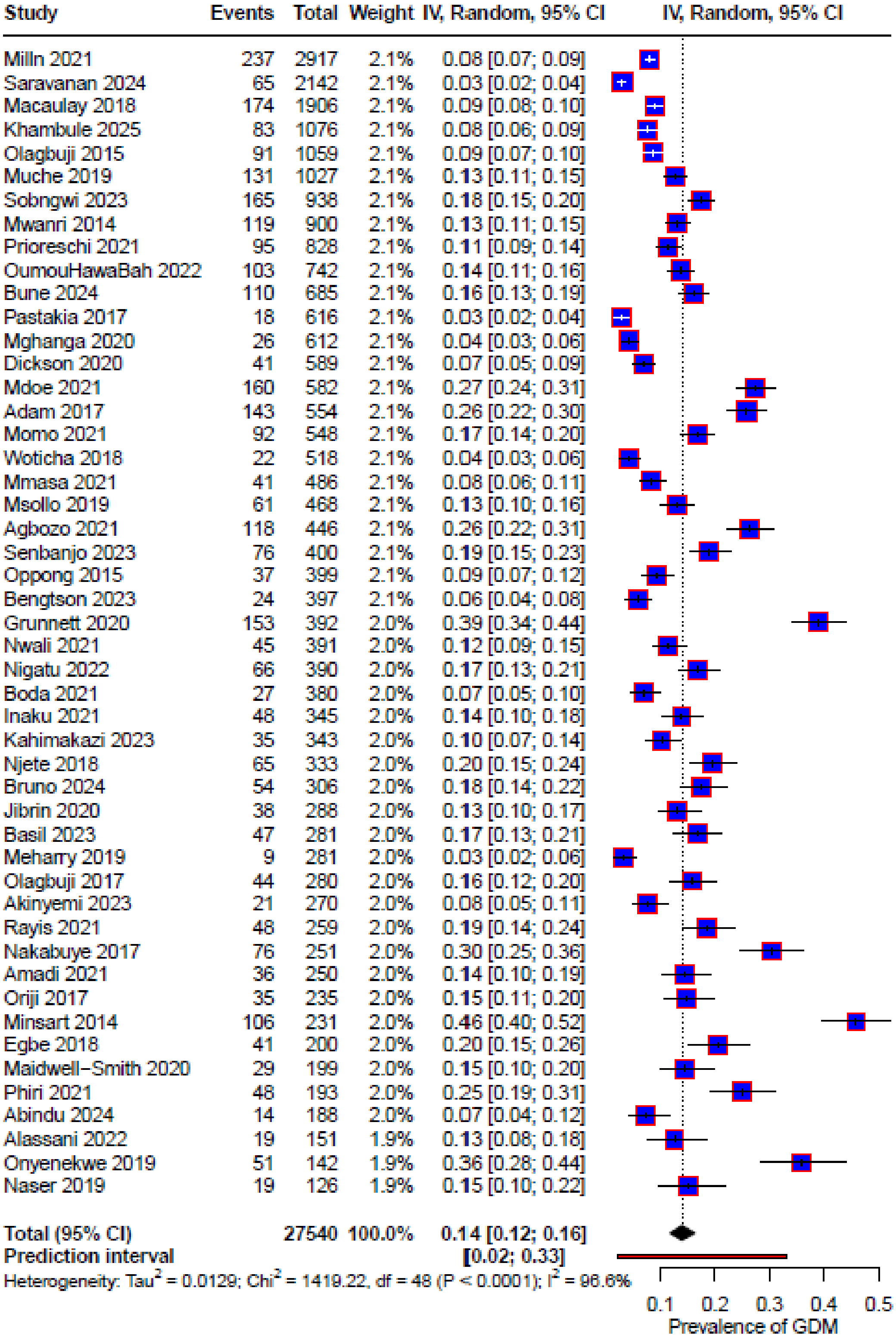

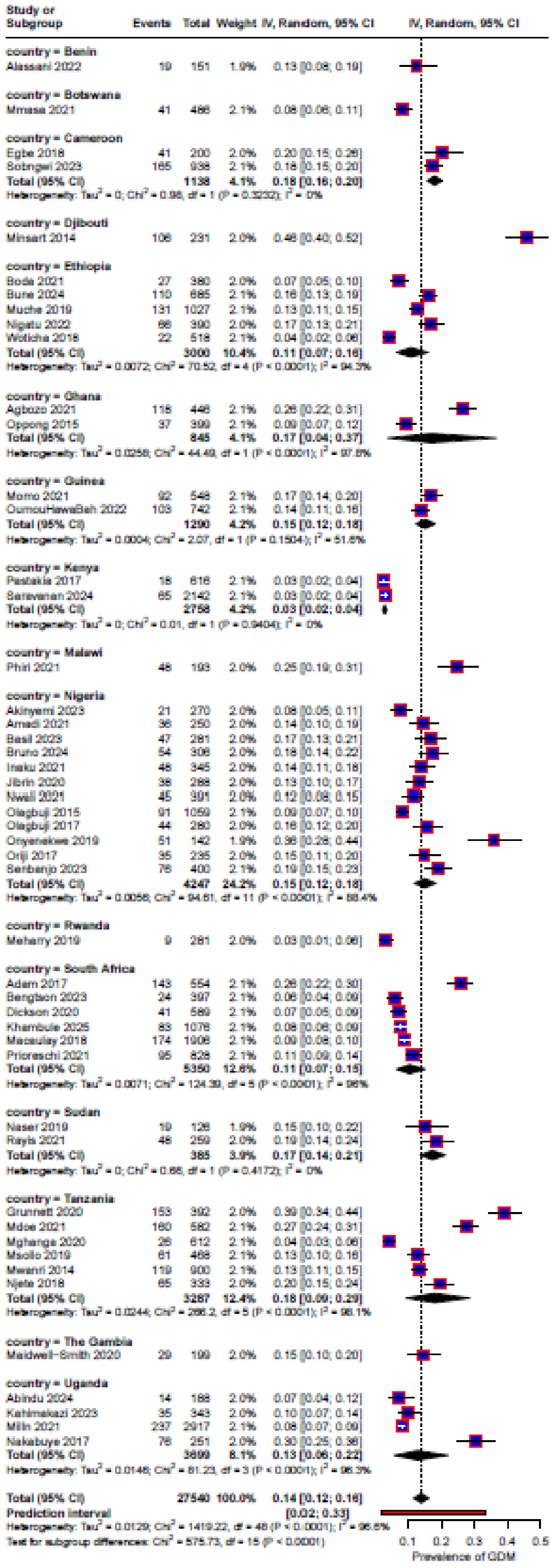
Study or Subgroup
Events
Total
Weight
IV, Random, 95% CI
IV, Random, 95% CI
country = Benin
Alassani 2022 19 151 1.9% 0.13 [0.08; 0.19]
country = Botswana
Mmasa 2021 41 486 2.1% 0.08 [0.06; 0.11]
country = Cameroon
Egbe 2018 41 200 2.0% 0.20 [0.15; 0.26]
Sobngwi 2023 165 938 2.1% 0.18 [0.15; 0.20]
Total (95% CI) 1138 4.1% 0.18 [0.16; 0.20]
Heterogeneity: Tau² = 0; Chi² = 0.98, df = 1 (P = 0.3232); I² = 0%
country = Djibouti
Minsart 2014 106 231 2.0% 0.46 [0.40; 0.52]
country = Ethiopia
Boda 2021 27 380 2.0% 0.07 [0.05; 0.10]
Bune 2024 110 685 2.1% 0.16 [0.13; 0.19]
Muche 2019 131 1027 2.1% 0.13 [0.11; 0.15]
Nigatu 2022 66 390 2.0% 0.17 [0.13; 0.21]
Woticha 2018 22 518 2.1% 0.04 [0.02; 0.06]
Total (95% CI) 3000 10.4% 0.11 [0.07; 0.16]
Heterogeneity: Tau² = 0.0072; Chi² = 70.52, df = 4 (P < 0.0001); I² = 94.3%
country = Ghana
Agbozo 2021 118 446 2.1% 0.26 [0.22; 0.31]
Oppong 2015 37 399 2.1% 0.09 [0.07; 0.12]
Total (95% CI) 845 4.1% 0.17 [0.04; 0.37]
Heterogeneity: Tau² = 0.0258; Chi² = 44.49, df = 1 (P < 0.0001); I² = 97.8%
country = Guinea
Momo 2021 92 548 2.1% 0.17 [0.14; 0.20]
OumouHawaBah 2022 103 742 2.1% 0.14 [0.11; 0.16]
Total (95% CI) 1290 4.2% 0.15 [0.12; 0.18]
Heterogeneity: Tau² = 0.0004; Chi² = 2.07, df = 1 (P = 0.1504); I² = 51.6%
country = Kenya
Pastakia 2017 18 616 2.1% 0.03 [0.02; 0.04]
Saravanan 2024 65 2142 2.1% 0.03 [0.02; 0.04]
Total (95% CI) 2758 4.2% 0.03 [0.02; 0.04]
Heterogeneity: Tau² = 0; Chi² = 0.01, df = 1 (P = 0.9404); I² = 0%
country = Malawi
Phiri 2021 48 193 2.0% 0.25 [0.19; 0.31]
country = Nigeria
Akinyemi 2023 21 270 2.0% 0.08 [0.05; 0.11]
Amadi 2021 36 250 2.0% 0.14 [0.10; 0.19]
Basil 2023 47 281 2.0% 0.17 [0.13; 0.21]
Bruno 2024 54 306 2.0% 0.18 [0.14; 0.22]
Inaku 2021 48 345 2.0% 0.14 [0.11; 0.18]
Jibrin 2020 38 288 2.0% 0.13 [0.10; 0.17]
Nwali 2021 45 391 2.0% 0.12 [0.08; 0.15]
Olagbuji 2015 91 1059 2.1% 0.09 [0.07; 0.10]
Olagbuji 2017 44 280 2.0% 0.16 [0.12; 0.20]
Onyenekwe 2019 51 142 1.9% 0.36 [0.28; 0.44]
Orji 2017 35 235 2.0% 0.15 [0.11; 0.20]
Senbanjo 2023 76 400 2.1% 0.19 [0.15; 0.23]
Total (95% CI) 4247 24.2% 0.15 [0.12; 0.18]
Heterogeneity: Tau² = 0.0056; Chi² = 94.61, df = 11 (P < 0.0001); I² = 88.4%
country = Rwanda
Meharry 2019 9 281 2.0% 0.03 [0.01; 0.06]
country = South Africa
Adam 2017 143 554 2.1% 0.26 [0.22; 0.30]
Bengtson 2023 24 397 2.1% 0.06 [0.04; 0.09]
Dickson 2020 41 589 2.1% 0.07 [0.05; 0.09]
Khambule 2025 83 1076 2.1% 0.08 [0.06; 0.09]
Macaulay 2018 174 1906 2.1% 0.09 [0.08; 0.10]
Prioreschi 2021 95 828 2.1% 0.11 [0.09; 0.14]
Total (95% CI) 5350 12.6% 0.11 [0.07; 0.15]
Heterogeneity: Tau² = 0.0071; Chi² = 124.39, df = 5 (P < 0.0001); I² = 96%
country = Sudan
Naser 2019 19 126 1.9% 0.15 [0.10; 0.22]
Rayis 2021 48 259 2.0% 0.19 [0.14; 0.24]
Total (95% CI) 385 3.9% 0.17 [0.14; 0.21]
Heterogeneity: Tau² = 0; Chi² = 0.66, df = 1 (P = 0.4172); I² = 0%
country = Tanzania
Grunnet 2020 153 392 2.0% 0.39 [0.34; 0.44]
Mdoe 2021 160 582 2.1% 0.27 [0.24; 0.31]
Mghanga 2020 26 612 2.1% 0.04 [0.03; 0.06]
Msollo 2019 61 468 2.1% 0.13 [0.10; 0.16]
Mwanri 2014 119 900 2.1% 0.13 [0.11; 0.15]
Njete 2018 65 333 2.0% 0.20 [0.15; 0.24]
Total (95% CI) 3287 12.4% 0.18 [0.09; 0.29]
Heterogeneity: Tau² = 0.0244; Chi² = 266.2, df = 5 (P < 0.0001); I² = 98.1%
country = The Gambia
Maidwell−Smith 2020 29 199 2.0% 0.15 [0.10; 0.20]
country = Uganda
Abindu 2024 14 188 2.0% 0.07 [0.04; 0.12]
Kahimakazi 2023 35 343 2.0% 0.10 [0.07; 0.14]
Milln 2021 237 2917 2.1% 0.08 [0.07; 0.09]
Nakabuye 2017 76 251 2.0% 0.30 [0.25; 0.36]
Total (95% CI) 3699 8.1% 0.13 [0.06; 0.22]
Heterogeneity: Tau² = 0.0146; Chi² = 81.23, df = 3 (P < 0.0001); I² = 96.3%
Total (95% CI) 27540 100.0% 0.14 [0.12; 0.16]
Prediction interval [0.02; 0.33]
Heterogeneity: Tau² = 0.0129; Chi² = 1419.22, df = 48 (P < 0.0001); I² = 96.6%
Test for subgroup differences: Chi² = 575.73, df = 15 (P < 0.0001)
0.1
0.2
0.3
0.4
0.5
Prevalence of GDM

Figure S2 – Baujat plot

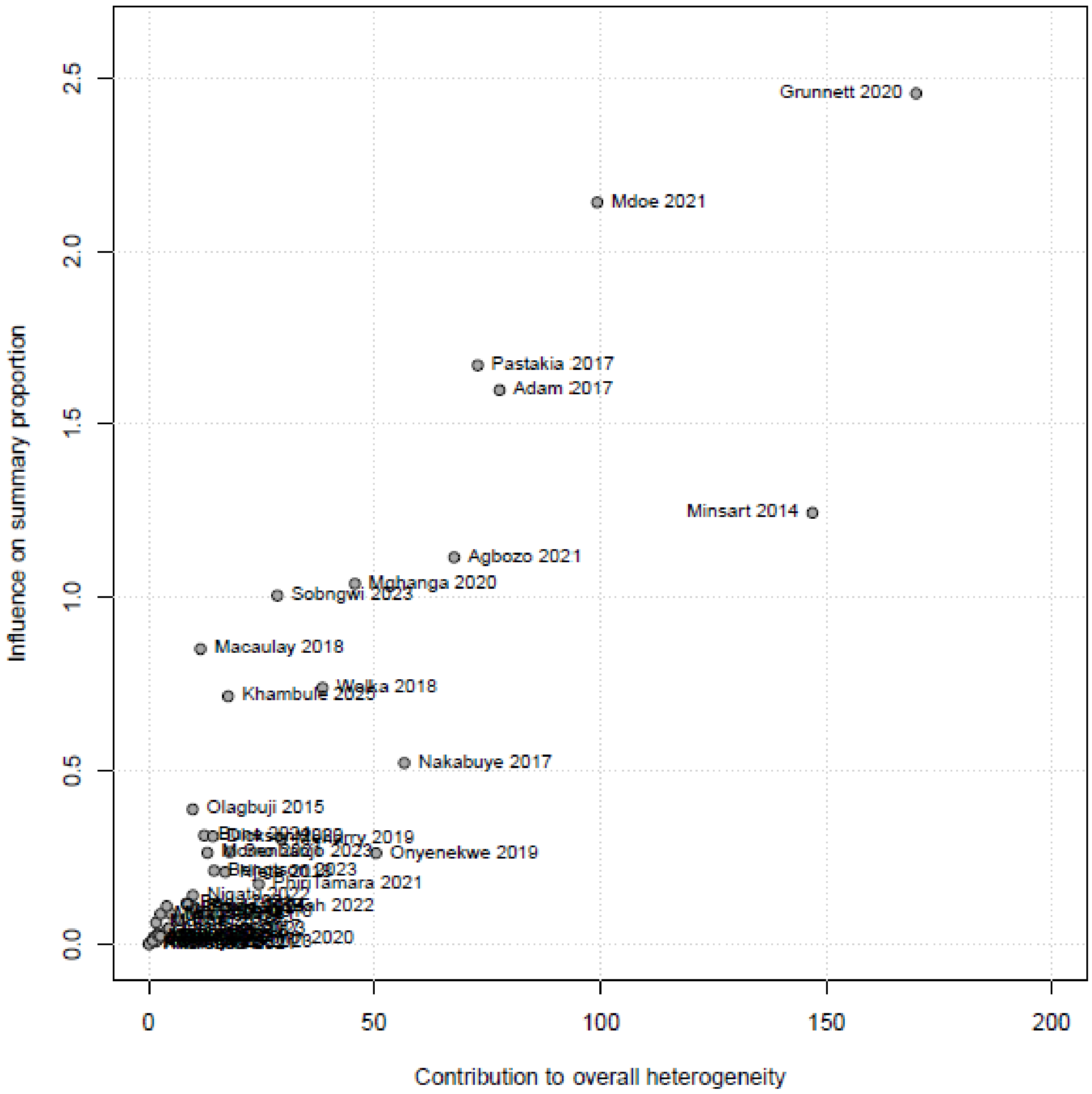

Thresholds for outlier investigation:

- Influence on summary proportion > 1.0
- Contribution to overall heterogeneity > 100

Text S3 – Sensitivity analysis data excluding sample size <300, use of POC tests and conducted prior to 2016

```
print(pes.large.lab.new)

   pred  ci.lb  ci.ub  pi.lb  pi.ub
 0.1093 0.0796 0.1429 0.0138 0.2768


> pes.subregion.sens <- metaprop(cases, size, label, data=rtest.large.lab.new, sm="
PFT", method.tau = "DL", method.ci = "NAsm", subgroup= subregion)
> summary(pes.subregion.sens)
                    proportion           95%-CI %W(common) %W(random) subregion
Khambule 2025           0.0771 [0.0619; 0.0939]        7.1        5.7     south
Bruno 2024              0.1765 [0.1357; 0.2213]        2.0        5.4      west
Bengtson 2023           0.0605 [0.0389; 0.0862]        2.6        5.5     south
Agbozo 2021             0.2646 [0.2246; 0.3066]        2.9        5.5      west
Nigatu 2022             0.1692 [0.1336; 0.2082]        2.6        5.4      east
Nwali 2021              0.1151 [0.0852; 0.1487]        2.6        5.4      west
Milln 2021              0.0812 [0.0716; 0.0914]       19.3        5.8      east
Mmasa 2021              0.0844 [0.0612; 0.1108]        3.2        5.5     south
Prioreschi 2021         0.1147 [0.0939; 0.1374]        5.5        5.6     south
Mghanga 2020            0.0425 [0.0278; 0.0600]        4.0        5.6      east
Macaulay 2018           0.0913 [0.0788; 0.1046]       12.6        5.7     south
Pastakia 2017           0.0292 [0.0172; 0.0442]        4.1        5.6      east
Adam 2017               0.2581 [0.2225; 0.2954]        3.7        5.6     south
Senbanjo 2023           0.1900 [0.1530; 0.2300]        2.6        5.5      west
Saravanan 2024          0.0303 [0.0235; 0.0381]       14.1        5.7      east
Dickson 2020            0.0696 [0.0504; 0.0917]        3.9        5.6     south
Inaku 2021              0.1391 [0.1045; 0.1778]        2.3        5.4      west
OumouHawaBah 2022       0.1388 [0.1148; 0.1647]        4.9        5.6      west

Number of studies: k = 18
Number of observations: o = 15143
Number of events: e = 1457

                     proportion           95%-CI
Common effect model      0.0888 [0.0843; 0.0934]
Random effects model     0.1092 [0.0817; 0.1399]

Quantifying heterogeneity (with 95%-CIs):
 tau^2 = 0.0097 [0.0058; 0.0259]; tau = 0.0985 [0.0762; 0.1609]
 I^2 = 96.9% [96.0%; 97.6%]; H = 5.68 [5.02; 6.43]

Test of heterogeneity:
      Q d.f.  p-value
 548.80   17 < 0.0001

Results for subgroups (common effect model):
                    k proportion           95%-CI      Q   I^2
subregion = south   7     0.0989 [0.0914; 0.1067] 125.69 95.2%
subregion = west    6     0.1662 [0.1522; 0.1807]  42.19 88.1%
subregion = east    5     0.0572 [0.0518; 0.0630] 130.09 96.9%

Test for subgroup differences (common effect model):
                    Q d.f.  p-value
```

```
Between groups 250.82    2 < 0.0001
Within groups  297.98   15 < 0.0001

Results for subgroups (random effects model):
                    k proportion           95%-CI  tau^2    tau
subregion = south   7     0.1022 [0.0683; 0.1420] 0.0063 0.0796
subregion = west    6     0.1681 [0.1280; 0.2124] 0.0043 0.0658
subregion = east    5     0.0622 [0.0311; 0.1031] 0.0069 0.0829

Test for subgroup differences (random effects model):
                   Q d.f. p-value
Between groups 13.63    2  0.0011

Details of meta-analysis methods:
- Inverse variance method
- DerSimonian-Laird estimator for tau^2
- Jackson method for confidence interval of tau^2 and tau
- Calculation of I^2 based on Q
- Freeman-Tukey double arcsine transformation
- Normal approximation confidence interval for individual studies
```

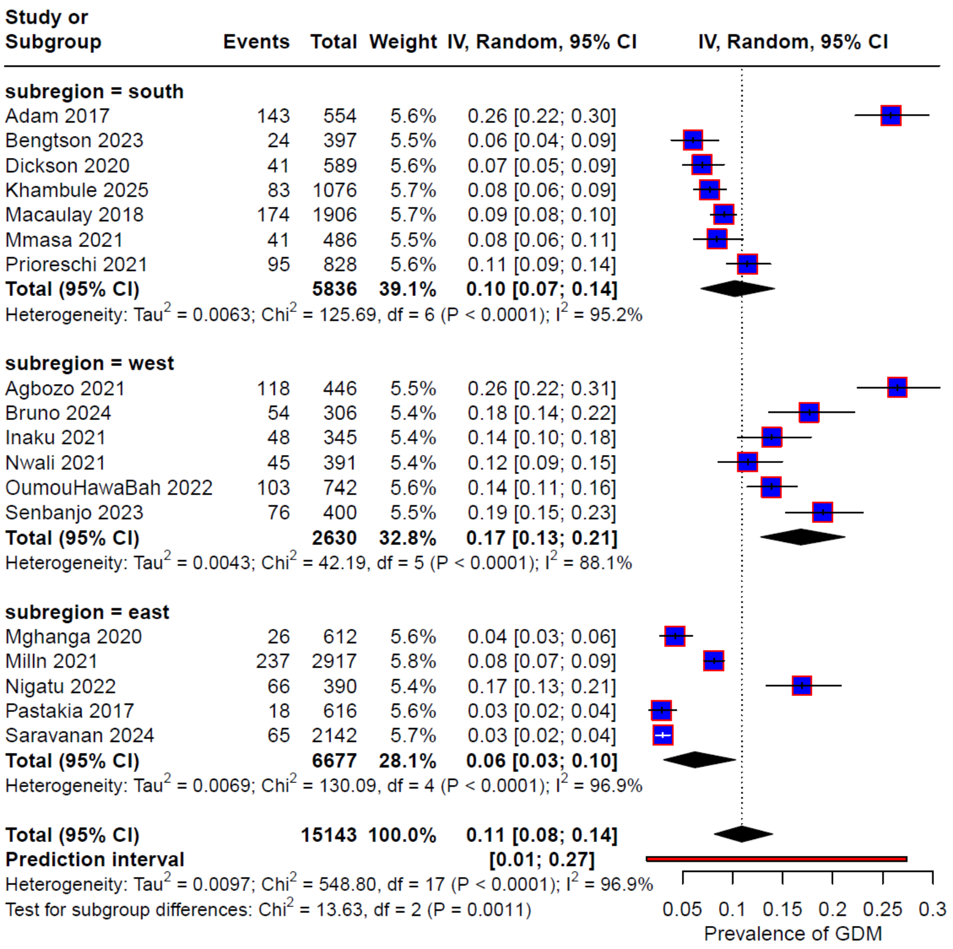

```
> metareg.largenewlab.moderators <- rma(yi, vi, data = ies.da.large.lab.new, mods =
~ subregion)
> summary(metareg.largenewlab.moderators)

Mixed-Effects Model (k = 18; tau^2 estimator: REML)

  logLik  deviance       AIC       BIC      AICc
 14.7097  -29.4194  -21.4194  -18.5872  -17.4194

tau^2 (estimated amount of residual heterogeneity):     0.0077 (SE = 0.0030)
tau (square root of estimated tau^2 value):             0.0880
I^2 (residual heterogeneity / unaccounted variability): 95.94%
H^2 (unaccounted variability / sampling variability):   24.63
R^2 (amount of heterogeneity accounted for):            32.93%

Test for Residual Heterogeneity:
QE(df = 15) = 297.9763, p-val < .0001

Test of Moderators (coefficients 2:3):
QM(df = 2) = 9.8054, p-val = 0.0074

Model Results:

                estimate      se    zval    pval    ci.lb   ci.ub
intrcpt           0.2535  0.0402  6.3114  <.0001   0.1748  0.3322  ***
subregionsouth    0.0729  0.0527  1.3842  0.1663  -0.0303  0.1761
subregionwest     0.1701  0.0548  3.1029  0.0019   0.0627  0.2775   **

---
Signif. codes:  0 '***' 0.001 '**' 0.01 '*' 0.05 '.' 0.1 ' ' 1
```

Text S4 – Sensitivity analysis data including non-IADPSG-equivalent diagnostic criteria

```
print(pes.othermethods)

   pred  ci.lb  ci.ub  pi.lb  pi.ub
 0.1317 0.1109 0.1540 0.0163 0.3300


metareg.moderators.othermethods <- rma(yi, vi, data = ies.da.othermethods, mods =
+                              ~ study_size + lab_test + sample_frame + criteria + study_age)
> summary(metareg.moderators.othermethods)

Mixed-Effects Model (k = 59; tau^2 estimator: REML)

  logLik  deviance       AIC       BIC      AICc
 34.0329  -68.0658  -34.0658   -4.1254   -9.5858

tau^2 (estimated amount of residual heterogeneity):     0.0114 (SE = 0.0026)
tau (square root of estimated tau^2 value):             0.1066
I^2 (residual heterogeneity / unaccounted variability): 95.44%
H^2 (unaccounted variability / sampling variability):   21.92
R^2 (amount of heterogeneity accounted for):            22.51%

Test for Residual Heterogeneity:
QE(df = 43) = 969.1005, p-val < .0001

Test of Moderators (coefficients 2:16):
QM(df = 15) = 31.2780, p-val = 0.0081

Model Results:

                        estimate      se     zval    pval    ci.lb    ci.ub
intrcpt                   0.3035  0.0613   4.9555  <.0001   0.1835   0.4236  ***
study_size0-299           0.0851  0.0415   2.0481  0.0406   0.0037   0.1665    *
study_size300-699         0.0155  0.0437   0.3548  0.7227  -0.0701   0.1011
lab_testpoc               0.1088  0.0398   2.7339  0.0063   0.0308   0.1868   **
lab_testunknown           0.0403  0.0553   0.7293  0.4658  -0.0680   0.1486
sample_frameLocal        -0.0241  0.0406  -0.5934  0.5529  -0.1036   0.0554
sample_framemulti-city   -0.1062  0.0525  -2.0224  0.0431  -0.2090  -0.0033    *
sample_frameregional      0.0142  0.0468   0.3027  0.7621  -0.0775   0.1059
criteriaCC               -0.1996  0.0786  -2.5392  0.0111  -0.3537  -0.0455    *
criteriaDIPSI            -0.0284  0.0711  -0.3995  0.6895  -0.1677   0.1109
criteriaIADPSGm          -0.0536  0.0557  -0.9638  0.3352  -0.1628   0.0555
criteriaWHO1985          -0.3357  0.1288  -2.6062  0.0092  -0.5881  -0.0832   **
criteriaWHO1999          -0.1218  0.0764  -1.5946  0.1108  -0.2716   0.0279
criteriaWHO2013          -0.0148  0.0390  -0.3803  0.7037  -0.0914   0.0617
study_ageold              0.0343  0.0503   0.6821  0.4952  -0.0643   0.1329
study_ageolder            0.1043  0.0635   1.6438  0.1002  -0.0201   0.2287

---
Signif. codes:  0 ‘***’ 0.001 ‘**’ 0.01 ‘*’ 0.05 ‘.’ 0.1 ‘ ’ 1
```
